# Oxidative stress affects sperm health and fertility—Time to apply facts learned at the bench to help the patient: Lessons for busy clinicians

**DOI:** 10.1002/rmb2.12598

**Published:** 2024-09-01

**Authors:** Pallav Sengupta, Germar‐M. Pinggera, Aldo E. Calogero, Ashok Agarwal

**Affiliations:** ^1^ Global Andrology Forum Moreland Hills Ohio USA; ^2^ Department of Biomedical Sciences, College of Medicine Gulf Medical University Ajman UAE; ^3^ Department of Urology Medical University Innsbruck Innsbruck Austria; ^4^ Division of Endocrinology, Metabolic Diseases and Nutrition University of Catania Catania Italy; ^5^ Cleveland Clinic Cleveland Ohio USA

**Keywords:** male infertility, oxidative stress, reactive oxygen species, sperm DNA fragmentation, sperm motility

## Abstract

**Background:**

Increased oxidative stress (OS), resulting from the delicate balance between reactive oxygen species (ROS) production and antioxidant defense, is closely linked to sperm abnormalities and male subfertility. Elevated ROS levels particularly affect sperm quality. The vulnerability of spermatozoa to ROS is due to the absence of DNA repair mechanisms and the high presence of polyunsaturated fatty acids in their membranes.

**Methods:**

This article updates and advances our understanding of the molecular damage caused by OS in spermatozoa, including lipid peroxidation, DNA damage, motility, and functionality. Additionally, the review discusses the challenges in diagnosing OS in semen and recommends accurate and sensitive testing methods. Case studies are utilized to demonstrate the effective management of male infertility caused by OS.

**Main findings:**

Highlighting the need to bridge the gap between research and clinical practice, this review suggests strategies for clinicians, such as lifestyle and dietary changes and antioxidant therapies. The review emphasizes lifestyle modifications and personalized care as effective strategies in managing male infertility caused by OS.

**Conclusion:**

This review calls for early detection and intervention and interdisciplinary collaboration to improve patient care in male infertility cases related to increased OS.

## Graphical abstract

Increased oxidative stress (OS), resulting from an imbalance between reactive oxygen species (ROS) production and antioxidant defense, is linked to sperm abnormalities and male subfertility. This article explores molecular damage in spermatozoa due to OS, emphasizing lipid peroxidation, DNA damage, motility, and functionality issues. It suggests clinical strategies, including lifestyle and dietary changes and antioxidant therapies, and underscores the importance of accurate diagnosis, early intervention, and interdisciplinary collaboration in managing male infertility related to OS.

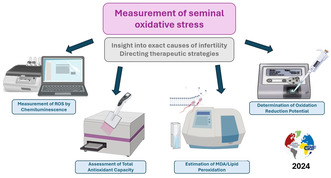

## 1 INTRODUCTION

Oxygen, while critical for the aerobic metabolism of spermatozoa, paradoxically contributes to the generation of reactive oxygen species (ROS), which are deleterious agents that can lead to oxidative stress (OS) and damage cellular structures.^1^ Studies have revealed an intricate relationship between ROS and sperm health, highlighting the pivotal role of elevated OS levels in various sperm abnormalities, including defects in the head, acrosome, midpiece, cytoplasmic droplets, and tail. Particularly in teratozoospermia, characterized by abnormal sperm morphology, elevated ROS levels are frequently implicated in subfertility or infertility.^2^, ^3^

In reproductive medicine, there remains a significant disconnect between the detailed laboratory research on OS and its effects on sperm health, and the actual use of this knowledge in clinical practice. Despite significant progress in identifying and treating male infertility, nearly 50% of cases remain unexplained, lacking a clear cause or contributing factor.^4^ This issue mainly arises from the difficulty in transforming laboratory‐based results into practical, patient‐focused treatments. The key challenge is to incorporate detailed molecular findings regarding OS and sperm function into concrete clinical procedures. This discrepancy highlights the urgent need for cross‐disciplinary teamwork, connecting research with clinical application, to improve fertility results using the deep understanding acquired through laboratory studies. Recent publications by the Global Andrology Forum (GAF) emphasize emerging trends and findings in the field of reproductive health, especially the role of OS in male infertility,^5^, ^6^ which strengthens the frontier of knowledge in this field. There is a need to synthesize recent research, highlight the ongoing challenges in translating these findings to therapeutic strategies, and propose actionable steps to integrate these insights into clinical settings. Thus, this review underscores the importance of understanding the delicate balance between ROS generation and antioxidant defense in male fertility, highlighting the need for translating bench research to clinical practice.

## 2 UNDERSTANDING OXIDATIVE STRESS

### 2.1 Basic concept: What is oxidative stress?

OS occurs when there is an imbalance between the production of ROS and the ability of the cellular antioxidant system to neutralize them.^7^ ROS are highly reactive molecules, typically generated during normal cellular metabolism, and can damage lipids, proteins, and nucleic acids. Excessive ROS production overwhelming cellular antioxidant defenses leads to increased OS, which is linked to various health issues, including male infertility.^8^, ^9^

ROS encompasses several forms, including oxygen free radicals like superoxide anions, hydroxyl radicals, and hyperoxyl radicals, along with non‐radical forms such as hypochlorous acid and hydrogen peroxide, as well as reactive nitrogen species. These factors play a multifaceted role in male fertility.^4^ At normal physiological levels, ROS are essential for sperm capacitation, the acrosome reaction, and sperm–egg fusion. Antioxidant defense in seminal fluid is crucial for maintaining sperm health by mitigating the harmful impacts of excessive ROS. This regulation is supported by antioxidants found in the seminal fluid, such as vitamins E and C, taurine, β‐mercaptoethanol, cysteine, cysteamine, and hypotaurine.^10^ However, when ROS levels surpass the scavenging capacity of these antioxidants, OS‐induced sperm damage occurs.

### 2.2 Sources of oxidative stress in the male reproductive system

In human ejaculate, the primary sources of ROS are seminal leukocytes and morphologically abnormal spermatozoa. Residual cytoplasm or cytoplasmic droplets, often containing enzymes like glucose‐6‐phosphate dehydrogenase (G6PD), significantly contribute to ROS production. Mitochondrial dysfunction and plasma membrane activity in spermatozoa, along with the enzyme NADPH oxidase 5 (NOX5), exacerbate ROS generation. Seminal fluid leukocytes, such as polymorphonuclear leukocytes and macrophages, are notable ROS producers, particularly when activated by infections or inflammation. Leukocytospermia, identified by the World Health Organization, is a condition marked by an abnormally high concentration of leukocytes in semen. Some of the important endogenous and exogenous sources accounting for OS in the male reproductive system are discussed below.

#### 2.2.1 Exogenous sources

##### Radiation

**FIGURE 1:**
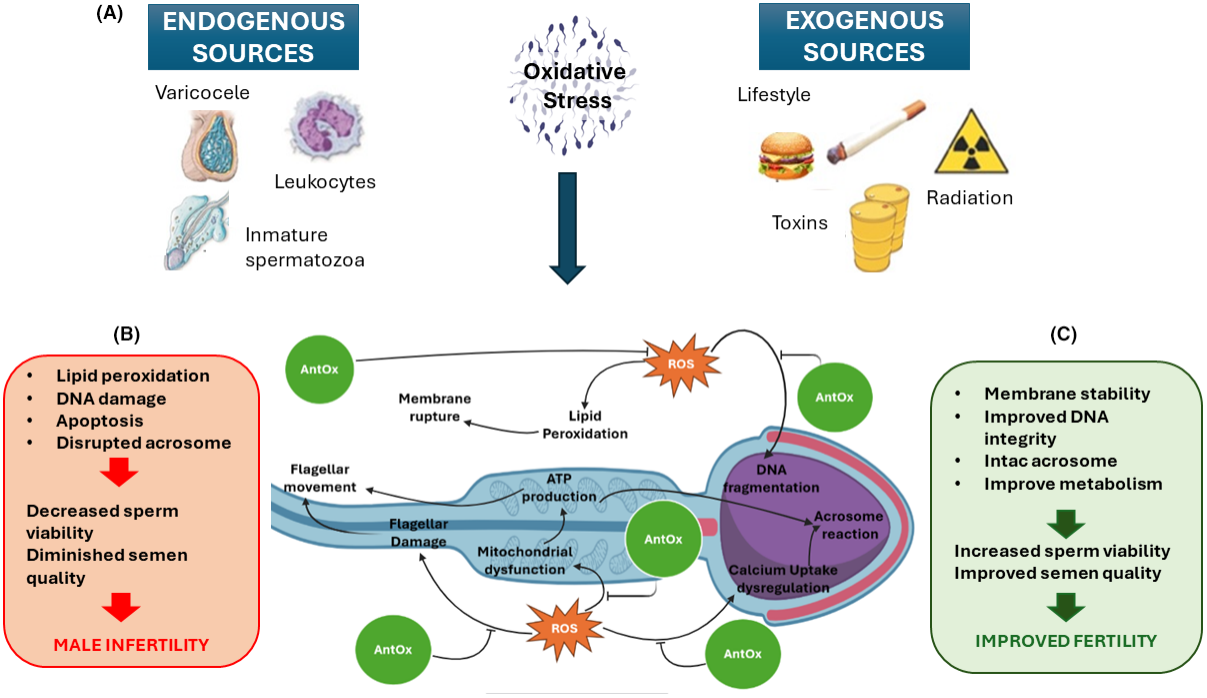
Impact of oxidative stress on male fertility. (A) Endogenous (varicocele, leukocytes, and immature spermatozoa) and exogenous (lifestyle, radiation, and toxins) sources of ROS lead to oxidative stress which results in (B) lipid peroxidation and DNA damage, resulting in decreased sperm viability and quality. (C) Antioxidants (AntOx) can mitigate these effects, enhancing membrane stability and sperm quality, thus improving fertility.

Mobile phone radiation significantly increases ROS in seminal plasma, damaging sperm DNA and affecting sperm motility, count, and vitality.^11^, ^12^ Both the thermal and non‐thermal effects of radiofrequency waves can disrupt spermatogenesis and induce sperm apoptosis (Figure 1).^13^, ^14^

##### Lifestyle factors

Smoking, a changeable lifestyle choice, substantially disrupts the balance between ROS production and antioxidant defenses, leading to increased seminal leukocyte and ROS levels of 48% and 107%, respectively.^15^, ^16^ Furthermore, smoking increases the concentrations of toxic elements such as lead and cadmium in semen and blood, enhancing ROS generation and adversely affecting sperm function.^13^ Alcohol consumption leads to increased acetaldehyde production, a byproduct of ethanol metabolism, further boosting ROS and reducing the percentage of functional spermatozoa.^13^, ^17^

##### Toxins

Increased industrial and domestic pollution introduces more environmental toxins and endocrine disruptors into the immediate surroundings, which can excessively stimulate testicular ROS production and thus OS, adversely affecting sperm morphology and function. Exposure to environmental toxins such as phthalates and heavy metals like lead and mercury is linked to reduced sperm count and quality.^18^, ^19^, ^20^

#### 2.2.2 Endogenous sources

##### Leukocytes

Peroxidase‐positive leukocytes, primarily polymorphonuclear leukocytes (50%–60%), and macrophages (20%–30%) are sourced from the seminal vesicles and prostate gland. During urogenital infections or inflammation, these cells can produce up to 100 times more ROS than usual, enhancing NADPH production through the hexose monophosphate shunt.^21^ Inflammation also increases pro‐inflammatory mediators concentrations and decreases antioxidant capacity, potentially triggering a respiratory burst leading to OS.^22^ Leukocytospermia is defined as the presence of over one million peroxidase‐positive leukocytes per milliliter of semen and is linked to significant impairments in sperm function (Figure 1).^23^

##### Immature spermatozoa

During normal sperm maturation of spermatids into mature, motile spermatozoa, excess cytoplasm is extruded by approximately 50%–75% of the total volume of the early spermatids. Disruption in this process results in the retention of excessive cytoplasm around the mid‐piece of spermatozoa, impairing their function (excess residual cytoplasm). Immature spermatozoa that retain cytoplasm and exhibit distorted head morphology are thus major contributors to seminal ROS.^24^ Excess cytoplasm harbors metabolic enzymes like glucose‐6‐phosphate dehydrogenase (G6PD) and NADPH oxidase, which are crucial for ROS production through NADPH.^25^ G6PD, in particular, is essential for catalyzing the hexose‐monophosphate shunt, facilitating ROS production and OS.^26^ Normal spermatozoa also produce ROS through NADPH oxidase (NOX5) in their plasma membrane and NAD(P)H‐dependent oxidoreductase (diaphorase) in their mitochondria, which is a key participant in the high‐energy Krebs cycle. This cycle primarily facilitates acetate oxidation, generating three NADH molecules from NAD+, which contribute to electron transport in mitochondria, producing a moderate amount of ROS.^27^, ^28^

##### Sertoli cells

Sertoli cells are capable of generating ROS.^29^, ^30^, ^31^ The addition of scavestrogens, synthetic steroidal estrogens with antioxidant properties, can inhibit ROS production in sertoli cells and mitigate iron‐induced cell damage.^31^, ^32^ This finding suggested that under normal conditions, sertoli cells may support spermatogenesis through controlled production of ROS.^31^

##### Varicocele

Varicocele, characterized by venous dilation in the pampiniform plexus with an abnormal blood flow, is a prevalent cause of male subfertility, affecting up to 40% of infertile male partners.^33^ It is believed to impair sperm function through testicular hyperthermia, toxic metabolites, and hypoxia, leading to OS.^9^, ^33^ Meta‐analyses have identified elevated levels of ROS and lipid peroxidation markers in semen from varicocele patients compared to healthy donors, with ROS levels correlating with varicocele severity.^33^, ^34^, ^35^

## 3 OXIDATIVE STRESS AND ITS IMPACT ON SPERM: UPDATED MECHANISMS

At physiological levels, ROS are necessary for processes such as sperm capacitation, acrosome reaction, and the fusion of spermatozoon and egg. The antioxidant defense in the seminal fluid is vital for preserving sperm health, as it counters the adverse effects of excess ROS. Many molecules and enzymatic systems have important scavenger effects.^10^ However, when ROS production exceeds the endogenous antioxidant capacity, the increased OS damages spermatozoa (Figure 1).

### 3.1 Lipid peroxidation and sperm membrane damage

Spermatozoa are uniquely susceptible to ROS‐induced damage. This vulnerability stems from several factors: the susceptibility of sperm chromatin condensation, the absence of DNA repair mechanisms in spermatozoa, high polyunsaturated fatty acid (PUFA) content in sperm membranes, ROS generation by spermatozoa (especially during the epididymal transit), limited cytoplasmic antioxidant enzymes in spermatozoa, and the prolonged duration spermatozoa spend in the male and female reproductive tracts.^36^, ^37^, ^38^, ^39^ Thus, OS leads to lipid peroxidation (LPO), wherein ROS targets the PUFAs in sperm membranes.^40^ This interaction results in changes in membrane fluidity, a decline in membrane integrity, and ultimately, impaired sperm function. The integrity of the sperm membrane is crucial as it influences sperm motility, an essential factor for successful fertilization.^41^ Multiple double bonds in PUFAs and a relative deficiency of cytoplasmic antioxidant enzymes in spermatozoa increase the susceptibility to OS.^12^ LPO is primarily initiated by the hydroxyl radical (·OH), which targets the vulnerable hydrogen–carbon bonds in the non‐conjugated double bonds of sperm membrane lipids, leading to the formation of stabilized free radicals that enhance lipid peroxidation susceptibility.^12^

Lipid peroxyl radicals propagate the chain reaction of lipid peroxidation by interacting with conjugated radicals, thus generating lipid hydroperoxides.^8^ This oxidative process impacts sperm function by oxidizing sulfhydryl groups, decreasing axonal protein phosphorylation, and reducing sperm motility. Furthermore, hydrogen peroxide, another form of ROS, can diffuse into spermatozoa and inhibit crucial metabolic enzymes such as glucose‐6‐phosphate dehydrogenase (G6PD). Inhibition of G6PD disrupts the pentose phosphate pathway, reducing the production of NADPH necessary for cellular reduction reactions.^12^ The diminished availability of NADPH impairs the activity of glutathione peroxidase, a critical antioxidant enzyme in spermatozoa that utilizes reduced glutathione to neutralize ROS. Consequently, a reduction in NADPH levels leads to an increase in phospholipid peroxidation, adversely affecting membrane fluidity and further decreasing sperm motility. Additionally, byproducts of lipid peroxidation, such as malondialdehyde (MDA), serve as biomarkers of oxidative damage in spermatozoa, and are detectable through various biochemical assays.^12^ The ROS‐induced electron loss from sperm membrane lipids further exacerbates LPO, producing mutagenic and genotoxic aldehydes like MDA, 4‐hydroxynonenal, and acrolein.^17^ Elevated ROS levels may also compromise mitochondrial membrane integrity, triggering caspase activation and subsequent apoptosis, thereby perpetuating ROS production, increasing DNA damage, and accelerating apoptotic processes.^42^ This cascade highlights the critical role of the sperm plasma membrane as a primary target for ROS, underscoring its potential to compromise genetic integrity through cascade signaling mechanisms.

### 3.2 Oxidative stress on semen parameters

OS plays a significant role in DNA damage within spermatozoa. ROS are known to cause both single‐ and double‐strand DNA breaks and chromatin crosslinking, leading to genetic anomalies.^43^, ^44^ Such DNA modifications not only reduce fertilization success rates but also pose risks of transmitting genetic defects to progeny, affecting the health of subsequent generations.^45^ A significant number of studies have underlined this correlation, providing a novel insight into the etiology of male infertility.^46^, ^47^ An elevated sperm DNA fragmentation (SDF) rate has been associated with reduced fertilization rates, poor embryo quality, lower pregnancy rates, and a higher risk of early pregnancy loss. An increasing body of evidence points to a robust correlation between seminal OS and SDF.^48^ One study showed that infertile patients with a high SDF also exhibited increased markers of OS, indicating an underlying link between these two parameters.^46^ Experimental models have demonstrated that exogenously induced OS leads to an increase in SDF, thereby directly substantiating this association.^49^, ^50^ Furthermore, interventional studies have shown that the reduction of seminal OS through antioxidant therapy leads to a decrease in the SDF rate, improving overall fertility outcomes. These studies provide compelling evidence for a positive correlation between seminal OS and SDF. Excessive ROS can inflict base modifications, strand breaks, and chromatin crosslinks, resulting in SDF.^45^, ^48^ However, the precise molecular pathways underlying this link require further investigation.

In relation to sperm motility and functionality, OS has been observed to adversely impact these critical attributes.^40^ Specifically, motility is affected by oxidative harm inflicted on the sperm tail and its energy source, thereby hindering its capability to progress toward and penetrate the oocyte.^51^ The spermatozoon, specifically its structural components such as the axoneme and the acrosome, exhibits a high susceptibility to oxidative damage instigated by ROS.^40^ The axoneme, which is essential for sperm motility and primarily composed of microtubules, is particularly vulnerable to OS. Elevated ROS levels can lead to lipid peroxidation of the membrane surrounding the axoneme, compromising its structural integrity and potentially altering its functionality. Such oxidative impairment can reduce sperm motility, thereby impeding the ability of the sperm to navigate the female reproductive tract to reach and fertilize the oocyte.^52^

Moreover, the acrosome reaction, which occurs in the acrosome located at the anterior region of the spermatozoon, is critical for penetration through the protective barriers of the oocyte. This reaction involves the release of enzymes essential for fertilization.^53^ However, OS can disrupt this finely tuned process by either prematurely triggering or completely inhibiting the acrosome reaction, consequently hindering effective adhesion and penetration of the oocyte.^54^ The dual role of ROS, as both essential signaling molecules and damaging agents, underscores the importance of maintaining a balanced oxidative state to preserve sperm functionality and optimize male reproductive potential.^14^

Comparative studies have consistently revealed that men with elevated OS levels exhibit markedly poorer sperm health than men with lower OS levels. This manifests as a decrease in sperm count, motility, and viability, and an increase in the SDF rate.^43^, ^51^, ^52^, ^55^, ^56^ The association between heightened OS and diminished sperm quality highlights the significance of maintaining an oxidative balance for optimal male reproductive health.

### 3.3 Genetic and epigenetic modifications

OS significantly affects both the genetic and epigenetic integrity of spermatozoa, which in turn influences early embryo development.^57^ This results in SDF, chromatin structural abnormalities, and a decline in overall sperm quality, including motility and fertilization potential.^48^ The epigenetic modifications induced by OS in spermatozoa are also crucial for understanding the developmental outcomes of the embryo.^58^, ^59^ Research has shown that spermatozoa exposed to oxidative conditions can lead to a significant developmental arrest at the stage of embryonic genome activation.^51^, ^60^ This process has been observed through various experimental studies, including those using animal models like cattle, where it was noted that embryos fertilized with spermatozoa exposed to OS displayed major developmental delays.^61^, ^62^ These changes in the sperm epigenetic landscape, such as modifications in DNA methylation patterns and histone configurations, do not necessarily correlate directly with the levels of DNA damage, indicating that the epigenetic reprogramming mechanisms might be independently sensitive to oxidative conditions.^57^, ^59^

Furthermore, the introduction of antioxidants has been suggested as a potential therapeutic approach to mitigate this oxidative damage, thus preserving both the genetic and epigenetic integrity necessary for successful fertilization and early embryo development.^63^ This finding suggests a pivotal role for targeted antioxidant therapies in improving reproductive outcomes in patients with OS‐induced infertility.

## 4 CLINICAL STUDIES AND RESEARCH DATA

### 4.1 Research studies

The OS plays a critical role in determining sperm quality and is intimately connected to the reproductive potential across various animal species. In numerous animal studies, particularly in marine invertebrates and mammals, there is mounting evidence that OS adversely impacts sperm functionality by inducing LPO and compromising mitochondrial integrity.^64^, ^65^, ^66^ In marine invertebrates like the ascidian *Ciona robusta* and the mussel *Mytilus galloprovincialis*, along with the mammal *Bos taurus*, studies have shown that higher ROS levels correlate with lower sperm motility.^64^, ^67^, ^68^, ^69^ This inverse relationship is often attributed to lipid peroxidation of sperm membranes. Oxidative damage reduces membrane fluidity, impacting sperm motility and ultimately its fertilizing capacity.^66^, ^70^ Moreover, LPO has been identified as a detrimental factor that decreases sperm quality by impairing its motility and vitality.^71^, ^72^ Mitochondrial functionality, which is essential for providing the energy necessary for sperm motility, is also affected by OS. Studies indicate that ROS can cause mitochondrial dysfunction by damaging the mitochondrial DNA, leading to decreased mitochondrial membrane potential (MMP) and is lower than the normal MMP by −80 to −120 mV and by altered electrochemical gradient to reduced ATP production, essential for all the energy‐dependent processes for sperm motility.^64^ In *B. taurus*, for instance, there is a positive correlation between MMP and sperm motility, suggesting that mitochondrial health is a critical determinant of motility and, by extension, fertilization capability.^73^ Interestingly, the response to OS and the resultant impact on sperm function appear to be species‐specific. While some species exhibit a direct negative impact of increased ROS on sperm quality, others show varying degrees of resilience or adaptation to oxidative conditions, which might reflect evolutionary adaptations to environmental OS.^64^, ^69^ For example, in *B. taurus*, the relationship between MMP and motility underscores the species‐specific energy metabolism strategies that spermatozoa employ to maintain functionality despite oxidative challenges.^69^, ^73^

In vitro studies on sperm quality and the adverse effects of OS have revealed significant impacts on sperm function and embryo development, with many studies conducted under controlled laboratory conditions.^74^, ^75^, ^76^ It has been reported how in vitro handling and manipulation of spermatozoa during assisted reproduction technology (ART) procedures can generate OS, which adversely affects sperm function. The research has indicated that spermatozoa experience increased ROS production during various in vitro procedures, such as washing, centrifugation, and cryopreservation. This OS is linked to sperm DNA damage, which can lead to reduced fertilization rates and compromised embryo development.^51^, ^76^ Furthermore, specific impacts observed in in vitro settings, such as decreased sperm motility and vitality due to oxidative modifications induced by handling and environmental stressors, have also been reported. For instance, the centrifugation process used in sperm preparation for intra‐cytoplasmic sperm injection (ICSI) and other ART techniques is particularly highlighted for exacerbating oxidative conditions, thereby increasing the likelihood of sperm DNA fragmentation. The resulting oxidative DNA damage in spermatozoa is critical because it holds potential implications for the success rates of ART outcomes, including lower pregnancy rates and increased risks of miscarriage.^51^, ^77^ These findings underscore the delicate balance required in in vitro environments to manage OS, highlighting the need for optimized protocols that minimize oxidative damage to spermatozoa, thereby preserving their functional integrity and enhancing the chances of successful fertilization and healthy embryo development. The ongoing challenge in ART‐related procedures is to refine and apply methods that reduce OS, such as antioxidant supplementation or gentler handling techniques, to improve overall reproductive outcomes.

Furthermore, omics studies in the realm of male fertility have illuminated the profound impacts of molecular mechanisms on sperm quality, offering a multidimensional understanding that extends beyond traditional assays. Through the comprehensive integration of omics datasets, Park et al.^78^ identified distinct molecular pathways governing male fertility in boars and bulls, highlighting species‐specific responses to fertility challenges. This study identified key differences in gamete production and protein biogenesis‐associated pathways in bulls with below‐normal fertility, suggesting a linkage between impaired protein synthesis during spermatogenesis and fertility outcomes. Conversely, boar spermatozoa with normal fertility exhibited enriched mitochondrial‐associated metabolic pathways, indicative of optimized energy metabolism contributing to better reproductive outcomes.^79^ Furthering the discourse, recent omics approaches have enabled the profiling of spermatozoa at an unprecedented scale, with studies identifying fertility‐related molecular markers that differentiate between varying fertility levels. These investigations not only enhance the understanding of sperm biology but also pave the way for novel diagnostic tools and therapeutic strategies aimed at improving male reproductive health. For instance, comparative omics analyses have highlighted the crucial role of mitochondrial functionality in sperm motility and overall fertility, demonstrating that the integrity of mitochondrial processes is critical for maintaining the energy supply required for effective sperm function and fertilization.^78^, ^80^ These insights from omics studies are reshaping the understanding of sperm quality and fertility, emphasizing the importance of molecular mechanisms in determining reproductive success. The integration of transcriptomic, proteomic, and metabolomic data offers a holistic view of the biological functions influencing sperm quality, which is vital for developing targeted interventions aimed at enhancing male fertility across different species.

### 4.2 Clinical studies

Excessive production of ROS occurs physiologically in several circumstances, including lifestyle factors—such as alcohol consumption, cigarette smoking, and obesity—or the presence of varicocele, exposure to radiation, taking medications, and so on.^81^ Various clinical studies have documented the negative impact of seminal OS on sperm quality and male fertility since Aitken and colleagues first reported ROS in washed human semen using a chemiluminescence assay.^82^

ROS can damage sperm ultrastructure leading to peroxidation of membrane lipids, proteins, and DNA, consequent to cellular apoptosis when its levels exceed the cellular scavenger capacity which is greatly reduced in spermatozoa.^21^ These events negatively influence sperm parameters, male fertility, and pregnancy outcomes.^24^, ^83^, ^84^, ^85^ This evidence has led to the coining of the acronym “MOSI” (male oxidative stress infertility), recently proposed to indicate those patients whose infertility is attributable to high levels of seminal OS.^86^ More specifically, a negative correlation between seminal OS levels and the percentage of spermatozoa with normal motility was outlined in a prospective clinical study on 39 infertile patients and 13 fertile controls. The high seminal OS has also emerged in patients with teratozoospermia with a higher percentage of spermatozoa with amorphous heads, damaged acrosomes, midsection defects, cytoplasmic remnants, and tail defects, suggesting that sperm morphology is a good indirect index of seminal OS.^83^ More recently, seminal OS was negatively correlated with sperm concentration and motility in a study of 847 patients.^87^

The high seminal OS has also been associated with poor ART outcomes and failure of embryo development in clinical settings.^88^ SDF, an indirect measure of the effects of OS on spermatozoa, has been associated with pregnancy outcomes. In particular, a meta‐analysis of 56 studies reported the negative impact of this parameter on the outcomes of ART (both IVF and ICSI).^89^ SDF has also been found to be associated with unexplained recurrent miscarriages (RPL)^90^ and the latest guidelines from the European Society for Human Reproduction and Embryology (ESHRE) on the management of RPL mention that SDF assessment can be considered for diagnostic purposes in couples with RPL.^91^

Despite sporadic attempts to find seminal ROS cut‐off values predictive of ART outcome,^92^ to date no threshold has been introduced into clinical practice, mainly due to measurement limitations (see Section 5.2 for details).

## 5 DIAGNOSING OXIDATIVE STRESS IN MALE INFERTILITY

### 5.1 Current methods for assessing oxidative stress in semen

#### 5.1.1 Semen analysis

The conventional analysis of sperm parameters, such as sperm count, morphology, and motility, offers clinicians a surrogate metric for evaluating seminal OS, with asthenozoospermia posited as a particularly reliable indicator of OS. An increase in seminal plasma viscosity is associated with elevated levels of MDA, a marker of lipid peroxidation, and a concomitant decrease in antioxidant capacity within the seminal plasma.^93^ Moreover, infections with *Ureaplasma urealyticum* in semen are linked to increased seminal plasma viscosity and enhanced generation of ROS.^93^ The presence of an excessive number of round cells in semen may suggest leukocytospermia, a known contributor to elevated ROS production. To differentiate these cells from immature spermatozoa, additional diagnostic assessments, such as peroxidase tests, seminal elastase measurement, or immunostaining for the cluster of differentiation 45 (CD45), a leukocyte‐specific transmembrane glycoprotein, are recommended. Notably, abnormal sperm morphology and the presence of cytoplasmic droplets are indicative of dysfunctional spermatozoa prone to unregulated ROS production. Furthermore, compromised integrity of the sperm membrane, assessable through the hypo‐osmotic swelling test, is associated with the presence of OS.^13^

#### 5.1.2 Total antioxidant capacity

To evaluate the total antioxidant capacity (TAC) within seminal plasma, luminol is utilized as a chemiluminescent probe. This assay is calibrated against Trolox, a water‐soluble analog of vitamin E, ensuring the standardization of measurements. The results are expressed in terms of a ROS‐TAC score, which quantifies the cumulative antioxidant activities contributed by all constituents, including vitamins, lipids, and proteins.^94^

#### 5.1.3 Evaluation of ROS via chemiluminescence

**FIGURE 2:**
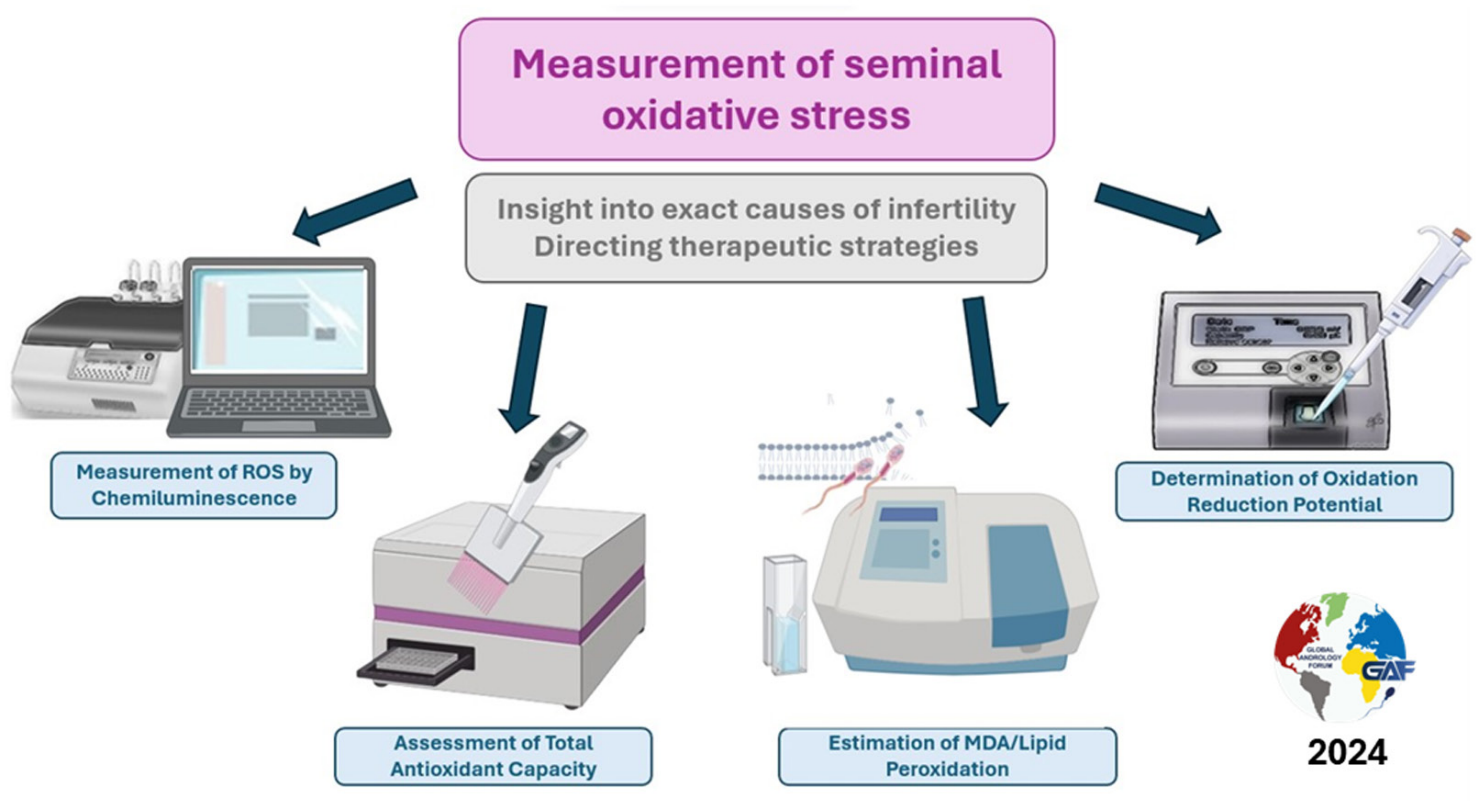
Different methods of measurement of seminal oxidative stress.

The quantification of ROS in seminal fluid is typically conducted using a chemiluminescence assay (Figure 2). This technique involves the utilization of a luminometer coupled with a chemiluminescent substrate, specifically luminal (5‐amino‐2,3‐dihydro‐1,4‐phthalazinedione; Sigma‐Aldrich, St. Louis, MO, USA). To prepare the samples, semen is initially liquefied and then centrifuged at 300 g for 7 min. The resultant seminal plasma is aliquoted and stored at −20°C for later TAC measurement. The remaining sperm pellet is washed with phosphate‐buffered saline (PBS, pH 7.4), and re‐suspended in PBS to a concentration of 2 × 10^6^ sperm/mL for the measurement of basal ROS levels. For the assay, a control reaction is set up using 10 mL of a 5 mM solution of luminol in 400 mL of PBS. Luminol, prepared as a 5 mM stock solution in dimethyl sulfoxide, is added to the sperm suspension to serve as the chemiluminescent probe. The reaction mixtures are then incubated within the luminometer for 15 min to facilitate the quantification of ROS levels. Luminol is sensitive to both extracellular and intracellular ROS, detecting these species through the emission of light upon reaction with the radicals. This emitted light is converted into an electrical (photon) signal by the luminometer, and the resultant data is expressed in relative light units per second per 10^6^ sperm. In assays involving washed sperm suspensions, normal ROS concentrations typically range from 0.10 to 1.03 × 10^6^ counted photons per minute per 20 × 10^6^ sperm.^94^

#### 5.1.4 Lipid peroxidation markers

In spermatozoa, the accumulation of lipid peroxides leads to the formation of various degradation products, notably MDA, acrolein, hydroxynonenal, and isoprostanes. These compounds serve as biomarkers of OS and can be quantitatively assessed.^95^ Among these biomarkers, MDA is most commonly measured using the thiobarbituric acid (TBA) assay. This assay exploits the interaction between MDA and TBA to form a 1:2 adduct, which is a colored complex. The concentration of this complex can be determined using fluorometric or spectrophotometric techniques.^13^, ^95^

#### 5.1.5 Oxidation–reduction potential in seminal fluid

The oxidation–reduction potential (ORP), also referred to as redox potential, quantifies the electron transfer capacity between chemical entities, encapsulating the dynamic balance between oxidants and reductants (Figure 2). This parameter is instrumental in assessing the OS within biological systems.^96^ Technological advancements have enabled the use of a galvanostatic method to monitor electron flux, which proves useful in gauging OS changes post‐trauma or during intense physical activity.^97^ Integrating ORP evaluation with conventional semen analysis aids in identifying the origins of poor semen quality and male infertility. The MiOXSYS System (Male Infertility Oxidative System; https://mioxsys.com/mioxsys‐system/), employing ultra‐high impedance electrometry, measures semen ORP by assessing electron exchange between antioxidants and oxidants present.^98^ Unlike alternative methodologies, the MiOXSYS technique requires neither specialized training for operation nor specific sample preparation protocols. It allows for the ORP determination from a minimal volume (30 μL) of fresh or thawed samples within approximately 4 min, maintaining result stability for up to 120 min post‐collection.^98^ If analysis post this timeframe is impractical, sample cryopreservation is recommended.

### 5.2 Challenges and limitations in the diagnosis

The introduction of the measurement of seminal ROS levels into clinical practice has been severely slowed down by the limitations of the tests currently in use. The sixth edition of the World Health Organization (WHO) semen analysis manual introduced ROS evaluation tests in the “advanced examination” section, which collects tests (e.g., luminol, ORP, and TAC) that do not have sufficient validation evidence and therefore are recommended only in a research context. Thus, their interpretation in the clinical setting requires a certain caution degree.^99^

Despite being the first method introduced for measuring ROS, chemiluminescence requires a lot of time and expensive equipment, and, above all, the results are highly variable. Sperm age, volume, centrifugation, temperature control, and background luminescence can interfere with the measurement, thus explaining the high intra‐individual variability in the test.^100^, ^101^ TAC has long been used to estimate the total antioxidant capacity, but is limited by the expensive equipment required, the time of inhibitory activity, and does not provide information on the levels of antioxidant enzymes that play an important scavenger role.^101^, ^102^ Tests for measuring MDA, an indirect indicator of high levels of OS at the seminal level, require rigorous controls, are not specific, and only provide post‐hoc measures.^101^

ORP has recently proven to be an attractive option for ROS assessment, amenable to standardization in the future. Some evidence suggests the reproducibility and reliability of the MiOXSYS in measuring ORP. ORP levels have been negatively correlated with sperm concentration, sperm motility, normal morphology, and total motile sperm count^103^ and positively with SDF rate.^46^, ^103^, ^104^ A cut‐off value of 1.34 (mV/10^6^ sperm/mL) was recently proposed to discriminate between good and poor‐quality sperm, with a positive predictive value of 94.7%.^98^, ^105^ However, sample viscosity can still pose a challenge, being a source of intra‐individual variability in ORP assessment.^101^

### 5.3 Recommendations for best practices in clinical settings

As indicated in the WHO manual for semen analysis,^99^ current tests for direct measurement of ROS levels should be avoided in clinical practice, until further validated in large multicenter cohort double‐blind studies. To date, the only test that has demonstrated sufficient reliability and reproducibility and has obtained the consensus of several companies are the tests that measure SDF, which represents an indirect measurement of seminal OS.

Accordingly, the SDF has been included in the latest edition of the WHO manual for semen analysis, in the “Extended examination” section, in which all tests that should not be performed routinely in clinical practice, but which can be required for diagnostic purposes in specific situations.^99^ Among all currently available tests for measuring SDF, the TUNEL test, the sperm chromatin dispersion test, the Comet test, and the acridine orange (AO) test are described in the manual.^99^

Overall, the guidelines of the main scientific societies such as the American Society for Reproductive Medicine (ASRM), the American Urological Association (AUA), the ESHRE, and the Italian Society of Andrology and Sexual Medicine (SIAMS), agree in not requiring the SDF test as a first‐level examination during the management of infertile patients.^91^, ^106^, ^107^ An orderly and sequential diagnostic process (of course starting from a detailed medical history) is essential for trying to understand the causes of infertility. The SDF test should be requested only in cases where conventional work‐up shows negative results and a clear etiology cannot be diagnosed. The ASRM/AUA and ESHRE suggest (while the European Urological Association [AUA] recommends) SDF testing in couples with RPL from natural conception or ART, as well as in men with unexplained infertility.^75^, ^91^, ^106^

## 6 TREATMENT APPROACHES TARGETING OXIDATIVE STRESS

### 6.1 Antioxidant therapy: Types, effectiveness, and potential risks

Antioxidants such as vitamins C and E have been shown to improve sperm quality by protecting spermatozoa from oxidative damage, thereby enhancing their motility.^108^, ^109^ Elements like coenzyme Q10 (CoQ10) and zinc also increase the success rates of fertilization in ART by bolstering sperm function.^110^, ^111^, ^112^, ^113^ Moreover, antioxidants help maintain the integrity of sperm DNA, reducing the risk of compromised embryo development and miscarriages.^114^ However, antioxidant supplementation is not without risks. Excessive intake can lead to pro‐oxidative effects, which increase OS and can harm sperm health.^115^ Some antioxidants may also interact with medications, potentially reducing their effectiveness or leading to adverse reactions.^116^ The optimal dosages of antioxidants for improving male reproductive health remain unclear, with both insufficient and excessive intakes posing potential risks.^116^, ^117^ The consumption of certain micronutrients and compounds has been thoroughly examined for their possible benefits in reducing OS in the male reproductive system.^118^

#### 6.1.1 l‐carnitine and acetyl‐l‐carnitine

l‐carnitine and acetyl‐l‐carnitine, both derivatives of the amino acid lysine, play crucial roles in the oxidation of mitochondrial fatty acids. In relation to male fertility, these compounds are vital for maintaining proper sperm morphology and motility.^119^, ^120^ High levels of ROS can impair sperm functionality. The administration of l‐carnitine and acetyl‐l‐carnitine has been shown to enhance sperm motility by reducing oxidative damage and improving mitochondrial function.^121^, ^122^

#### 6.1.2 Zinc and folic acid

Zinc, a vital trace mineral, is essential for a variety of physiological functions including DNA synthesis, RNA transcription, and cellular metabolism. Folic acid is key for DNA synthesis and repair. In male reproductive health, deficiencies in either nutrient can diminish sperm quality.^123^ The combined supplementation of zinc and folic acid has been shown to increase sperm count in men with reduced fertility, suggesting a synergistic effect that may protect against oxidative damage to sperm DNA.^124^

#### 6.1.3 Vitamin E and selenium

Vitamin E, a lipid‐soluble antioxidant, and selenium, a trace element, are both powerful antioxidants essential for preventing oxidative damage in sperm, thereby enhancing motility and overall sperm health. Their combined supplementation has shown greater efficacy in improving male reproductive health than taking either nutrient alone.^125^, ^126^

#### 6.1.4 Coenzyme Q10

CoQ10 is a critical component of the mitochondrial electron transport chain, essential for energy production, and also acts as an antioxidant.^112^, ^127^ Deficiency in CoQ10 can impair sperm motility due to reduced energy production and increased OS. Supplementation has been shown to improve sperm parameters by mitigating oxidative damage and enhancing energy production in spermatozoa.^111^, ^127^

Despite the favorable outcomes reported in numerous studies, some findings indicate minimal or no improvement in sperm parameters following antioxidant supplementation. These discrepancies may be due to differences in study designs, sample populations, types and dosages of antioxidants, and duration of supplementation.^116^ As researchers continue to explore potential treatments for male infertility, they find increasing evidence that antioxidants and dietary modifications can play significant role in improving reproductive health and fertility outcomes. They offer a practical approach to combating OS, a significant detriment to male reproductive health. However, careful application is essential. Extensive, ongoing research is needed to determine optimal antioxidant amounts and combinations, and understanding individual responses to these treatments will be crucial for tailoring personalized therapies. While the associated risks are generally low compared to the potential benefits, medical approaches should be based on thorough diagnostic evaluations and scientifically sound guidelines.^128^

### 6.2 Lifestyle and dietary modifications

Research has demonstrated that improvements in semen quality can be achieved through targeted dietary changes and regular exercise, independent of body mass index changes. Enhancements include better sperm concentration, motility, morphology, and reduced DNA fragmentation.^129^, ^130^, ^131^ Animal studies further suggest that these lifestyle changes can positively affect embryo development and offspring metabolic health.^132^ Adhering to Mediterranean‐style diets—rich in fruits, vegetables, seafood, and antioxidant‐laden plant foods—also correlates with superior semen quality.^133^, ^134^

In terms of micronutrients, carotenes, ascorbic acid, tocopherols, selenium, zinc, l‐arginine, and CoQ10 are particularly beneficial for male fertility.^135^ Moderate exercise is advisable for improving fertility and mitigating OS, though intense exercise may be detrimental.^136^ Eliminating tobacco use significantly enhances sperm parameters,^137^, ^138^, ^139^ and alcohol intake should be minimal, with no more than 5 units per week to maintain optimal fertility.^140^, ^141^, ^142^ Caffeine should be limited to the equivalent of 3 cups of coffee daily,^143^ and cannabis use is discouraged for managing male infertility.^144^ The potentially harmful effects of anabolic steroids on the male hypothalamic–pituitary–testicular axis could involve the use of gonadotropins, selective estrogen receptor modulators, and aromatase inhibitors. However, the use of these substances in an off‐label manner is not well‐researched.^145^

Effective management of psychological stress through meditation, yoga, and similar practices can improve male fertility.^146^, ^147^ Further investigation is necessary to define the precise benefits of stress reduction techniques and therapeutic approaches like cognitive behavioral therapy. It is also vital to manage stress related to sexual performance to enhance fertility outcomes. Sufficient sleep appears to play a crucial role in enhancing semen quality, as suggested by research.^148^, ^149^ Nonetheless, the specific lifestyle parameters and their optimal levels remain undefined and warrant additional research.

### 6.3 Emerging therapies and future directions in treatment

Emerging therapies and future directions in the treatment of OS in male reproductive health focus on advancing current methodologies and exploring innovative approaches.^150^ The continuous development of more targeted antioxidant therapies is a prime area of interest. Novel antioxidants and compounds that specifically target mitochondrial function and reduce ROS production are under investigation.^151^, ^152^ These could offer more precise mechanisms for protecting spermatozoa against oxidative damage. Additionally, gene therapy presents a promising frontier. Research is aiming to correct genetic defects that contribute to increased OS or compromised antioxidant defenses in spermatozoa.^150^, ^153^ Techniques such as CRISPR/Cas9 offer the potential for directly repairing these genetic anomalies, thereby enhancing sperm quality and overall reproductive health.^153^ Nanotechnology is another emerging field that could play a significant role in treating male infertility related to OS. Nanoparticles can be engineered to deliver antioxidants directly to specific cells or tissues, potentially increasing the efficacy and reducing the side effects associated with the systemic administration of antioxidants.^154^, ^155^ Furthermore, the role of the microbiome in male reproductive health is gaining attention. Studies suggest that modulating the gut microbiome could influence systemic antioxidant levels and immune responses, indirectly impacting OS levels and fertility.^156^ As the understanding of the biochemical pathways involved in male fertility deepens, personalized medicine approaches are becoming more feasible. These would involve comprehensive genomic, proteomic, and metabolomic profiling to tailor specific antioxidant therapies to individual needs, enhancing both effectiveness and safety.^157^ Thus, while antioxidants and lifestyle modifications currently offer significant benefits in managing OS in male reproductive health, the field is evolving. Future therapies are likely to be more precise and personalized, addressing the underlying causes of OS with greater accuracy and fewer side effects. Extensive research and clinical trials will be essential to validate these innovative approaches and integrate them into standard practice. However, ethical considerations of genetic diagnostics and emerging technologies, like CRISPR, include potential long‐term effects, unintended consequences, and psychological impacts. Responsible integration requires preventing misuse, ensuring informed consent, equitable access, and psychological support.

## 7 CASE STUDIES SHOWCASING SUCCESS IN IMPROVING SPERM HEALTH AFTER REDUCING OXIDATIVE STRESS

The patient, a 32‐year‐old male, presented with his 28‐year‐old partner to the fertility clinic with concerns about difficulty conceiving. The couple had been trying to conceive for the past 18 months without success. The male partner reported a generally healthy lifestyle (Mediterranean diet, regular physical activity, and no alcohol or drug use) but admitted to experiencing high levels of stress at work as a software engineer in a high‐stress environment. He referred to smoking 10 cigarettes a day for 10 years. He denied any history of significant medical conditions, including diabetes mellitus, and had not undergone any surgeries. Uneventful physical and genital examination revealed normal‐sized and firm testes (right testicular volume: 20 mL and left testicular volume: 18 mL), and no varicocele, hydrocele, or other abnormalities were confirmed via high‐resolution ultrasound.

The laboratory test results included a normal complete blood count, liver and kidney function test results, and normal endocrinologic assessments, including insulin resistance.

In two repeated semen analyses, there was a mild oligoasthenoteratozoospermia without leukocytospermia but elevated levels of SDF rate at the TUNEL test (10%), with local laboratory cut‐off normal values <4% and elevated ROS in semen according to the MiOXSYS test, with a value of −5.7 mV/10^6^ spermatozoa/mL. The patient was therefore diagnosed with MOSI in the absence of other identifiable causes. His management plan consisted of lifestyle modification with counseling on stress management techniques and advised to quit smoking. The prescribed medical treatment consisted of daily antioxidants (including vitamin C, vitamin E, carnitine, Zn and Se, and CoQ10) together with dietary modifications to include antioxidant‐rich foods. In the follow‐up semen analysis after 3 and 6 months, there was an improvement in conventional semen parameters, SDF rate, and normalization of the MiOXSYS value to 1.2 mV/10^6^ spermatozoa/mL.

This case highlights the impact of OS on male fertility, particularly in the context of a modern lifestyle characterized by high stress and suboptimal habits. OS can lead to sperm dysfunction by damaging the sperm membrane and DNA, resulting in decreased fertility. Management focuses on identifying and mitigating contributing factors, antioxidant therapy, and supportive care.

## 8 LINKING BENCH RESEARCH TO CLINICAL PRACTICE

### 8.1 Highlighting key takeaways that can be applied in clinical settings

OS is a well‐established cause of male infertility due to its adverse effects on sperm health and male fertility.^158^, ^159^, ^160^, ^161^, ^162^ According to Mayorga‐Torres, increased intracellular ROS production and DNA fragmentation have been observed in infertile patients compared to fertile men, whereas no significant differences were observed in conventional sperm parameters between fertile men and infertile patients. Furthermore, OS‐induced DNA damage in spermatozoa of male infertile patients may have implications for the health of children conceived in vitro.^163^

### 8.2 Interventions and solutions for busy clinicians

Addressing male infertility related to high levels of ROS and therefore OS is a nuanced area in which andrologists can play a pivotal role. The intervention strategies include two fundamental aspects, eliminating or reducing as much as possible all the causes of increased OS and increasing the levels of substances with antioxidant activity.

As regards the first aspect, interventions must be aimed at eliminating, where possible, all diseases that cause an increase in OS (e.g., urogenital infections, obesity, varicocele, etc.) and lifestyle changes. The latter can be recommended to the patient at the time of the first visit and includes stopping cigarette smoking, drinking alcohol, using narcotics, and so on. The patient should also be advised to avoid, if possible, occupational exposure to toxins that can increase ROS levels (e.g., industrial chemicals, pesticides, etc.).

As regards the second aspect, the patient can be advised, after careful evaluation of his diet, to increase the intake of foods richer in antioxidants. Along this same line, the opportunity for treatment with antioxidants should also be discussed with the patient. It has indeed been shown that the prescription of these supplements improves sperm quality and patient fertility by reducing OS, although with a low level of evidence.

It is essential to choose the molecule/s and the dosage of antioxidants based on the results of the laboratory tests performed on the patient so that the prescription is as compliant as possible with the pathophysiological aspects present in the patient. Indeed, it is useful to remember that although antioxidants are effective if appropriately prescribed, individual responses can vary significantly.

Therefore, the treatment strategy should be personalized based on a thorough assessment of the patient's general health, lifestyle, and specific fertility concerns. Doctors should also stay up to date on the latest research and clinical guidelines in this evolving field to provide the best possible care for their patients.

### 8.3 Diagnostic tests available for measuring oxidative stress in semen samples

The assessment of OS in semen samples is a critical aspect of male infertility diagnosis, hence for a successful treatment. However, the current diagnostic tests have limitations (see Section 5.2).

Castleton^164^ reported that the MiOXSYS® and OxiSperm® II assays, while included in the WHO manual, did not provide additional clinical utility beyond standard semen analysis. Overall, the absence of significant associations between nitroblue tetrazolium (NBT)‐reactivity and measurements of sperm function or OS suggests the limited diagnostic potential of the MiOXSYS and OxiSperm II assays. Agarwal^165^ suggested that the ORP test could be a cost‐efficient and sensitive option for measuring OS in semen. Tunc^166^ developed a standardized protocol for the NBT assay, which is effective in identifying sperm OS. Gosalvez^158^ highlighted the need for an inexpensive and easy‐to‐perform assay to detect OS in semen.

Overall, the measurement of OS in the semen fluid is of great relevance for a proper diagnosis. However, further research is of pivotal importance in this area.

### 8.4 Recommendations for reducing oxidative stress

In the realm of reproductive health and male infertility, the use of antioxidant supplements is a topic of significant interest, especially in cases where high ROS production and hence increased OS have been diagnosed. Excessive weight has been linked to reduced sperm production but also to higher OS. Therefore, diet and daily exercise need to be planned appropriately. A deficiency of nutrients, particularly zinc, selenium, and vitamin C, may disturb sperm production. Therefore, it is important to have a healthy and balanced diet. Proper treatment following the doctor's instructions and daily exercise boosts the immune system and normalizes the situation.^167^ Furthermore, infection, inflammation, and other diseases eventually present and capable of increasing the levels of OS must be treated with their specific therapeutic approaches. Supplementation can be used if the diet lacks the required amounts of nutrients with antioxidant properties. Currently, despite the effectiveness of antioxidant administration in improving conventional sperm parameters and pregnancy rate,^6^ there is no generally accepted agreement on the best supplementation therapy, either as a single compound or as a mixture of them. Furthermore, the level of evidence of the various studies published in the literature is classified as low or moderate quality, due to the lack of standardized therapeutic regimens widely used in these studies and the lack of common inclusion criteria for the male population undergoing treatment.^6^ The acceptance of antioxidant supplements for treating male infertility varies globally. In some countries, these supplements are widely used and recommended, while in others, they are prescribed with more caution due to a lack of comprehensive and well‐designed clinical trials. Regulatory agencies like the FDA in the United States or the EMA in Europe have different standards and guidelines for supplement use, which impacts global acceptance. Insert here the results and ref of the GAF Survey. However, it is crucial to note that the efficacy and safety of these supplements can vary and over‐supplementation can sometimes have adverse effects.

### 8.5 Importance of early detection and intervention

Understanding the role of ROS in male infertility and recognizing specific OS markers will enable clinicians to tailor treatments that target the underlying oxidative damage. This potentially results in reversing sperm abnormalities and increasing the chances of successful conception. Early intervention in patients with high ROS levels addresses immediate fertility issues, as it enables targeted interventions such as lifestyle modifications and antioxidant therapy, to overcome OS. However, accurate diagnostic methods also help prevent long‐term reproductive health complications, emphasizing the importance of routine screening for OS markers in male fertility assessments. This is particularly important in cases of male subfertility or idiopathic infertility, and probably even more given a history of RPL.

### 8.6 Encouraging interdisciplinary collaboration between research scientists and clinicians for optimal patient care

Encouraging interdisciplinary collaboration between researchers and clinicians is vital to optimize patient care in cases of male infertility attributed to high OS, as this collaboration fosters the integration of cutting‐edge scientific insights with clinical expertise, leading to better outcomes and personalized treatment strategies. In particular, the standardization of reliable and reproducible tests to measure OS is urgently needed. This problem can be solved and rapidly introduced into clinical practice with continued strong collaboration between basic scientists and clinicians.

## 9 FUTURE DIRECTIONS IN RESEARCH AND CLINICAL PRACTICE

The future directions in OS research and clinical practice involve several critical advancements and shifts in focus to enhance male reproductive health. A significant area of future research will likely be the development and integration of advanced diagnostic tools that can accurately and non‐invasively assess OS levels in semen. Such tools will be crucial for the early detection of oxidative damage, allowing for timely interventions that could significantly improve male fertility outcomes. Additionally, studies using omics technologies to uncover new biomarkers and therapeutic targets are expected to further elucidate the molecular pathways influenced by OS.

On the clinical front, personalized medicine will become increasingly important. Treatments tailored to individual OS profiles and genetic backgrounds are expected to significantly improve patient outcomes. This approach will leverage insights gained from advanced genomics and proteomics studies, enabling clinicians to design antioxidant therapies that are more effective and have fewer side effects than current options. Furthermore, interdisciplinary collaboration between researchers, clinicians, and technologists will be essential to translate these findings from bench to bedside rapidly and efficiently. The integration of artificial intelligence and machine learning in diagnostic and treatment processes could also play a transformative role, offering new ways to manage and treat OS‐related male infertility.

## 10 CONCLUSION

This review has underscored the pivotal role that OS plays in male infertility, providing clinicians with a deeper understanding of how bench research translates into clinical practice. Key takeaways for clinicians include the importance of early detection and management of OS, as highlighted by the molecular intricacies and pathological consequences discussed. Clinicians are encouraged to adopt advanced diagnostic tools and consider antioxidant therapies alongside lifestyle and dietary modifications to improve patient outcomes. The integration of bench research into clinical settings, particularly in the field of male fertility, has the potential to significantly enhance patient care. This review not only bridges the gap between theoretical research and practical application but also emphasizes the necessity for ongoing interdisciplinary collaborations. Such endeavors will enable the development of targeted therapies that mitigate oxidative stress and improve sperm quality, thus addressing the underlying causes of male infertility and enhancing reproductive outcomes.

## CONFLICT OF INTEREST STATEMENT

The authors declare no conflict of interest.

## References

[1] Kobayashi CI , Suda T . Regulation of reactive oxygen species in stem cells and cancer stem cells. J Cell Physiol. 2012;227(2):421–430. 10.1002/jcp.22764 21448925

[2] Rato L , Alves MG , Socorro S , Duarte AI , Cavaco JE , Oliveira PF . Metabolic regulation is important for spermatogenesis. Nat Rev Urol. 2012;9(6):330–338. 10.1038/nrurol.2012.77 22549313

[3] Ammar O , Mehdi M , Muratori M . Teratozoospermia: its association with sperm DNA defects, apoptotic alterations, and oxidative stress. Andrology. 2020;8(5):1095–1106. 10.1111/andr.12778 32096605

[4] Ghuman N , Ramalingam M . Male infertility. Obstet Gynaecol Reprod Med. 2018;28(1):7–14.

[5] Gül M , Russo GI , Kandil H , Boitrelle F , Saleh R , Chung E , et al. Male infertility: new developments, current challenges, and future directions. World J Men's Health. 2024;42:502–517. 10.5534/wjmh.230232 38164030 PMC11216957

[6] Agarwal A , Aa F , Saleh R , Hamoda TA , Harraz AM , Kavoussi P , et al. Controversy and consensus on indications for sperm DNA fragmentation testing in male infertility: a global survey, current guidelines, and expert recommendations. World J Men's Health. 2023;41(3):575–602. 10.5534/wjmh.220282 37118960 PMC10307662

[7] Agarwal A , Sengupta P . Oxidative stress and its association with male infertility. In: Parekattil S , Esteves S , Agarwal A , editors. Male infertility: contemporary clinical approaches, andrology, ART and antioxidants. Cham: Springer; 2020. p. 57–68. 10.1007/978-3-030-32300-4_6

[8] Saleh RA , Agarwal A . Oxidative stress and male infertility: from research bench to clinical practice. J Androl. 2002;23(6):737–752. 10.1002/j.1939-4640.2002.tb02324.x 12399514

[9] Makker K , Agarwal A , Sharma R . Oxidative stress & male infertility. Indian J Med Res. 2009;129(4):357–367. 19535829

[10] Serafini S , O'Flaherty C . Redox regulation to modulate phosphorylation events in human spermatozoa. Antioxid Redox Signal. 2022;37(7–9):437–450. 10.1089/ars.2021.0117 34714121

[11] Agarwal A , Deepinder F , Sharma RK , Ranga G , Li J . Effect of cell phone usage on semen analysis in men attending infertility clinic: an observational study. Fertil Steril. 2008;89(1):124–128. 10.1016/j.fertnstert.2007.01.166 17482179

[12] Aitken RJ , Gibb Z , Baker MA , Drevet J , Gharagozloo P . Causes and consequences of oxidative stress in spermatozoa. Reprod Fertil Dev. 2016;28(2):1–10. 10.1071/RD15325 27062870

[13] Agarwal A , Virk G , Ong C , du Plessis SS . Effect of oxidative stress on male reproduction. World J Men's Health. 2014;32(1):1–17. 10.5534/wjmh.2014.32.1.1 24872947 PMC4026229

[14] Dutta S , Majzoub A , Agarwal A . Oxidative stress and sperm function: a systematic review on evaluation and management. Arab J Urol. 2019;17:87–97. 10.1080/2090598X.2019.1599624 31285919 PMC6600059

[15] Agarwal A , Desai NR , Ruffoli R , Carpi A . Lifestyle and testicular dysfunction: a brief update. Biomed Pharmacother. 2008;62(8):550–553. 10.1016/j.biopha.2008.07.052 18771892

[16] Durairajanayagam D . Lifestyle causes of male infertility. Arab J Urol. 2018;16(1):10–20. 10.1016/j.aju.2017.12.004 29713532 PMC5922227

[17] Bui A , Sharma R , Henkel R , Agarwal A . Reactive oxygen species impact on sperm DNA and its role in male infertility. Andrologia. 2018;50(8):e13012. 10.1111/and.13012 29644708

[18] Wang M , Su P . The role of the Fas/FasL signaling pathway in environmental toxicant‐induced testicular cell apoptosis: an update. Syst Biol Reprod Med. 2018;64(2):93–102. 10.1080/19396368.2017.1422046 29299971

[19] Sengupta P , Nwagha U , Dutta S , Krajewska‐Kulak E , Izuka E . Evidence for decreasing sperm count in African population from 1965 to 2015. Afr Health Sci. 2017;17(2):418–427. 10.4314/ahs.v17i2.16 29062337 PMC5637027

[20] Sengupta P . Environmental and occupational exposure of metals and their role in male reproductive functions. Drug Chem Toxicol. 2013;36(3):353–368. 10.3109/01480545.2012.710631 22947100

[21] Agarwal A , Saleh RA , Bedaiwy MA . Role of reactive oxygen species in the pathophysiology of human reproduction. Fertil Steril. 2003;79(4):829–843. 10.1016/S0015-0282(02)04948-8 12749418

[22] Ford WC , Whittington K , Williams AC . Reactive oxygen species in human sperm suspensions: production by leukocytes and the generation of NADPH to protect sperm against their effects. Int J Androl. 1997;20(Suppl 3):44–49. 9466185

[23] Press W . Laboratory manual for the examination and processing of human semen. Geneva: World Health Organization; 2010. p. 7–113.

[24] Agarwal A , Tvrda E , Sharma R . Relationship amongst teratozoospermia, seminal oxidative stress and male infertility. Reprod Biol Endocrinol. 2014;12(1):45. 10.1186/1477-7827-12-45 24884815 PMC4049374

[25] Aitken RJ , Fisher HM , Fulton N , Gomez E , Knox W , Lewis B , et al. Reactive oxygen species generation by human spermatozoa is induced by exogenous NADPH and inhibited by the flavoprotein inhibitors diphenylene iodonium and quinacrine. Mol Reprod Dev. 1997;47(4):468–482. 10.1002/(SICI)1098-2795(199708)47:4<468::AID-MRD14>3.0.CO;2-S 9211432

[26] Rengan AK , Agarwal A , van der Linde M , du Plessis SS . An investigation of excess residual cytoplasm in human spermatozoa and its distinction from the cytoplasmic droplet. Reprod Biol Endocrinol. 2012;10:92. 10.1186/1477-7827-10-92 23159014 PMC3551780

[27] Gavella M , Lipovac V . NADH‐dependent oxidoreductase (diaphorase) activity and isozyme pattern of sperm in infertile men. Arch Androl. 1992;28(2):135–141. 10.3109/01485019208987691 1520038

[28] Du Plessis SS , Agarwal A , Halabi J , Tvrda E . Contemporary evidence on the physiological role of reactive oxygen species in human sperm function. J Assist Reprod Genet. 2015;32(4):509–520. 10.1007/s10815-014-0425-7 25646893 PMC4380893

[29] Gelain DP , Cammarota M , Zanotto‐Filho A , de Oliveira RB , Dal‐Pizzol F , Izquierdo I , et al. Retinol induces the ERK1/2‐dependent phosphorylation of CREB through a pathway involving the generation of reactive oxygen species in cultured Sertoli cells. Cell Signal. 2006;18(10):1685–1694. 10.1016/j.cellsig.2006.01.008 16510265

[30] Zanotto‐Filho A , Schröder R , Moreira JCF . Xanthine oxidase‐dependent ROS production mediates vitamin A pro‐oxidant effects in cultured sertoli cells. Free Radic Res. 2008;42(6):593–601. 10.1080/10715760802144422 18569017

[31] Hipler U , Görnig M , Hipler B , Römer W , Schreiber G . Stimulation and scavestrogen‐induced inhibition of reactive oxygen species generated by rat sertoli cells. Arch Androl. 2000;44(2):147–154. 10.1080/014850100262326 10746872

[32] Stanczyk FZ . Estrogens: different types and properties. In: Menopause. San Diego: Academic Press; 2000. p. 421–428.

[33] Agarwal A , Prabakaran S , Allamaneni SS . Relationship between oxidative stress, varicocele and infertility: a meta‐analysis. Reprod Biomed Online. 2006;12(5):630–633. 10.1016/S1472-6483(10)61190-X 16790111

[34] Cho CL , Esteves SC , Agarwal A . Novel insights into the pathophysiology of varicocele and its association with reactive oxygen species and sperm DNA fragmentation. Asian J Androl. 2016;18(2):186–193. 10.4103/1008-682X.170441 26732105 PMC4770484

[35] Will MA , Swain J , Fode M , Sonksen J , Christman GM , Ohl D . The great debate: varicocele treatment and impact on fertility. Fertil Steril. 2011;95(3):841–852. 10.1016/j.fertnstert.2011.01.002 21272869 PMC3046876

[36] Agarwal A , Makker K , Sharma R . Clinical relevance of oxidative stress in male factor infertility: an update. Am J Reprod Immunol. 2008;59(1):2–11. 10.1111/j.1600-0897.2007.00559.x 18154591

[37] Lewis S , Aitken R . DNA damage to spermatozoa has impacts on fertilization and pregnancy. Cell Tissue Res. 2005;322:33–41. 10.1007/s00441-005-1097-5 15912407

[38] Chenoweth PJ . Influence of the male on embryo quality. Theriogenology. 2007;68(3):308–315. 10.1016/j.theriogenology.2007.04.002 17482670

[39] Prakash S , Prithiviraj E , Suresh S , Lakshmi NV , Ganesh MK , Anuradha M , et al. Morphological diversity of sperm: a mini review. Iran J Reprod Med. 2014;12(4):239. 24976817 PMC4071627

[40] Sabeti P , Pourmasumi S , Rahiminia T , Akyash F , Talebi AR . Etiologies of sperm oxidative stress. Int J Reprod Biomed. 2016;14(4):231–240. 27351024 PMC4918773

[41] Collodel G , Moretti E , Noto D , Corsaro R , Signorini C . Oxidation of polyunsaturated fatty acids as a promising area of research in infertility. Antioxidants. 2022;11(5):1002. 10.3390/antiox11051002 35624866 PMC9137497

[42] Wagner H , Cheng JW , Ko EY . Role of reactive oxygen species in male infertility: an updated review of literature. Arab J Urol. 2018;16(1):35–43. 10.1016/j.aju.2017.11.001 29713534 PMC5922220

[43] Bibov MY , Kuzmin AV , Alexandrova AA , Chistyakov VA , Dobaeva NM , Kundupyan OL . Role of the reactive oxygen species induced DNA damage in human spermatozoa dysfunction. AME Med J. 2018;3(1):1–12. 10.21037/amj.2018.01.06

[44] Cho CL , Agarwal A . Role of sperm DNA fragmentation in male factor infertility: a systematic review. Arab J Urol. 2018;16(1):21–34. 10.1016/j.aju.2017.11.002 29713533 PMC5922225

[45] Panner Selvam MK , Sengupta P , Agarwal A . Sperm DNA fragmentation and male infertility. In: Arafa M , Elbardisi H , Majzoub A , Agarwal A , editors. Genetics of male infertility: a case‐based guide for clinicians. Cham: Springer; 2020. p. 155–172. 10.1007/978-3-030-37972-8_9

[46] Majzoub A , Arafa M , Mahdi M , Agarwal A , Al Said S , Al‐Emadi I , et al. Oxidation–reduction potential and sperm DNA fragmentation, and their associations with sperm morphological anomalies amongst fertile and infertile men. Arab J Urol. 2018;16(1):87–95. 10.1016/j.aju.2017.11.014 29713539 PMC5922185

[47] Dorostghoal M , Kazeminejad S , Shahbazian N , Pourmehdi M , Jabbari A . Oxidative stress status and sperm DNA fragmentation in fertile and infertile men. Andrologia. 2017;49(10):e12762. 10.1111/and.12762 28124476

[48] Agarwal A , Majzoub A , Baskaran S , Selvam MKP , Cho CL , Henkel R , et al. Sperm DNA fragmentation: a new guideline for clinicians. World J Men's Health. 2020;38(4):412–471. 10.5534/wjmh.200128 32777871 PMC7502318

[49] Kumar TR , Doreswamy K , Shrilatha B . Oxidative stress associated DNA damage in testis of mice: induction of abnormal sperms and effects on fertility. Mutat Res. 2002;513(1–2):103–111. 10.1016/S1383-5718(01)00300-X 11719095

[50] La Maestra S , De Flora S , Micale RT . Effect of cigarette smoke on DNA damage, oxidative stress, and morphological alterations in mouse testis and spermatozoa. Int J Hyg Environ Health. 2015;218(1):117–122. 10.1016/j.ijheh.2014.08.006 25260855

[51] Gualtieri R , Kalthur G , Barbato V , Longobardi S , Di Rella F , Adiga SK , et al. Sperm oxidative stress during in vitro manipulation and its effects on sperm function and embryo development. Antioxidants. 2021;10(7):1025. 10.3390/antiox10071025 34202126 PMC8300781

[52] Nowicka‐Bauer K , Nixon B . Molecular changes induced by oxidative stress that impair human sperm motility. Antioxidants. 2020;9(2):134. 10.3390/antiox9020134 32033035 PMC7070831

[53] Dahan T , Breitbart H . Involvement of metabolic pathway in the sperm spontaneous acrosome reaction. Theriogenology. 2022;192:38–44. 10.1016/j.theriogenology.2022.08.018 36044805

[54] El‐Taieb MA , Ali MA , Nada EA . Oxidative stress and acrosomal morphology: a cause of infertility in patients with normal semen parameters. Middle East Fertil Soc J. 2015;20(2):79–85. 10.1016/j.mefs.2014.05.003

[55] Agarwal A , Panner Selvam MK , Baskaran S , Cho CL . Sperm DNA damage and its impact on male reproductive health: a critical review for clinicians, reproductive professionals and researchers. Expert Rev Mol Diagn. 2019;19(6):443–457. 10.1080/14737159.2019.1614916 31056976

[56] Nguyen ND , Le MT , Dang HNT , Van Nguyen T , Nguyen QHV , Cao TN . Impact of semen oxidative stress on sperm quality: initial results from Vietnam. J Int Med Res. 2023;51(8):03000605231188655. 10.1177/03000605231188655 37572034 PMC10423449

[57] Menezo YJ , Silvestris E , Dale B , Elder K . Oxidative stress and alterations in DNA methylation: two sides of the same coin in reproduction. Reprod Biomed Online. 2016;33(6):668–683. 10.1016/j.rbmo.2016.09.006 27742259

[58] Bashiri Z , Amidi F , Amiri I , Zandieh Z , Maki CB , Mohammadi F , et al. Male factors: the role of sperm in preimplantation embryo quality. Reprod Sci. 2021;28:1788–1811. 10.1007/s43032-020-00334-z 33140326

[59] Breton‐Larrivée M , Elder E , McGraw S . DNA methylation, environmental exposures and early embryo development. Anim Reprod. 2019;16:465–474. 10.21451/1984-3143-ar2019-0062 32435290 PMC7234019

[60] De Castro LS , De Assis PM , Siqueira AF , Hamilton TR , Mendes CM , Losano JD , et al. Sperm oxidative stress is detrimental to embryo development: a dose‐dependent study model and a new and more sensitive oxidative status evaluation. Oxid Med Cell Longev. 2016;2016:8213071. 10.1155/2016/8213071 26770658 PMC4684862

[61] Bittner L , Wyck S , Herrera C , Siuda M , Wrenzycki C , Van Loon B , et al. Negative effects of oxidative stress in bovine spermatozoa on in vitro development and DNA integrity of embryos. Reprod Fertil Dev. 2018;30(10):1359–1368. 10.1071/RD17533 29712617

[62] Wyck S , Herrera C , Requena CE , Bittner L , Hajkova P , Bollwein H , et al. Oxidative stress in sperm affects the epigenetic reprogramming in early embryonic development. Epigenetics Chromatin. 2018;11:1–17. 10.1186/s13072-018-0224-y 30333056 PMC6192351

[63] Zarbakhsh S . Effect of antioxidants on preimplantation embryo development in vitro: a review. Zygote. 2021;29(3):179–193. 10.1017/S0967199420000660 33441217

[64] Gallo A , Esposito MC , Tosti E , Boni R . Sperm motility, oxidative status, and mitochondrial activity: exploring correlation in different species. Antioxidants. 2021;10(7):1131. 10.3390/antiox10071131 34356364 PMC8301117

[65] Pintus E , Ros‐Santaella JL . Impact of oxidative stress on male reproduction in domestic and wild animals. Antioxidants. 2021;10(7):1154. 10.3390/antiox10071154 34356386 PMC8301082

[66] Rojas Mora A , Meniri M , Glauser G , Vallat A , Helfenstein F . Badge size reflects sperm oxidative status within social groups in the House Sparrow Passer domesticus. Front Ecol Evol. 2016;4:67. 10.3389/fevo.2016.00067

[67] Gallo A , Boni R , Buttino I , Tosti E . Spermiotoxicity of nickel nanoparticles in the marine invertebrate Ciona intestinalis (ascidians). Nanotoxicology. 2016;10(8):1096–1104. 10.1080/17435390.2016.1177743 27080039 PMC4975092

[68] Gallo A , Tosti E . Adverse effect of antifouling compounds on the reproductive mechanisms of the ascidian Ciona intestinalis. Mar Drugs. 2013;11(9):3554–3568. 10.3390/md11093554 24065165 PMC3806468

[69] Boni R , Gallo A , Montanino M , Macina A , Tosti E . Dynamic changes in the sperm quality of *Mytilus galloprovincialis* under continuous thermal stress. Mol Reprod Dev. 2016;83(2):162–173. 10.1002/mrd.22604 26663619

[70] Chandra AK , Sengupta P , Goswami H , Sarkar M . Effects of dietary magnesium on testicular histology, steroidogenesis, spermatogenesis and oxidative stress markers in adult rats. Indian J Exp Biol. 2013;51(1):37–47. 23441478

[71] Kadirvel G , Kumar S , Kumaresan A . Lipid peroxidation, mitochondrial membrane potential and DNA integrity of spermatozoa in relation to intracellular reactive oxygen species in liquid and frozen‐thawed buffalo semen. Anim Reprod Sci. 2009;114(1–3):125–134. 10.1016/j.anireprosci.2008.10.002 19010614

[72] Chandra AK , Sengupta P , Goswami H , Sarkar M . Excessive dietary calcium in the disruption of structural and functional status of adult male reproductive system in rat with possible mechanism. Mol Cell Biochem. 2012;364:181–191. 10.1007/s11010-011-1217-3 22262485

[73] Leite RF , de Agostini Losano JD , Kawai GKV , Rui BR , Nagai KK , Castiglioni VC , et al. Sperm function and oxidative status: effect on fertility in *Bos taurus* and *Bos indicus* bulls when semen is used for fixed‐time artificial insemination. Anim Reprod Sci. 2022;237:106922. 10.1016/j.anireprosci.2022.106922 35065462

[74] Almansa‐Ordonez A , Bellido R , Vassena R , Barragan M , Zambelli F . Oxidative stress in reproduction: a mitochondrial perspective. Biology. 2020;9(9):269. 10.3390/biology9090269 32899860 PMC7564700

[75] Nikitaras V , Zander‐Fox D , McPherson NO . Improving sperm oxidative stress and embryo quality in advanced paternal age using idebenone in vitro—a proof‐of‐concept study. Antioxidants. 2021;10(7):1079. 10.3390/antiox10071079 34356315 PMC8301200

[76] Al‐Saleh I , Coskun S , Al‐Rouqi R , Al‐Rajudi T , Eltabache C , Abduljabbar M , et al. Oxidative stress and DNA damage status in couples undergoing in vitro fertilization treatment. Reprod Fertil. 2021;2(2):117–139. 10.1530/RAF-20-0062 35128448 PMC8812407

[77] Mauchart P , Vass RA , Nagy B , Sulyok E , Bódis J , Kovács K . Oxidative stress in assisted reproductive techniques, with a focus on an underestimated risk factor. Curr Issues Mol Biol. 2023;45(2):1272–1286. 10.3390/cimb45020083 36826028 PMC9954903

[78] Park YJ , Pang WK , Pang MG . Integration of omics studies indicates that species‐dependent molecular mechanisms govern male fertility. J Anim Sci Biotechnol. 2023;14(1):28. 10.1186/s40104-023-00836-1 36859388 PMC9979430

[79] Chen X , Zhu H , Hu C , Hao H , Zhang J , Li K , et al. Identification of differentially expressed proteins in fresh and frozen‐thawed boar spermatozoa by iTRAQ‐coupled 2D LC‐MS/MS. Reproduction. 2014;147(3):321–330. 10.1530/rep-13-0313 24357664

[80] Samanta L , Parida R , Dias TR , Agarwal A . The enigmatic seminal plasma: a proteomics insight from ejaculation to fertilization. Reprod Biol Endocrinol. 2018;16:1–11. 10.1186/s12958-018-0358-6 29704899 PMC5923003

[81] Takeshima T , Usui K , Mori K , Asai T , Yasuda K , Kuroda S , et al. Oxidative stress and male infertility. Reprod Med Biol. 2021;20(1):41–52. 10.1002/rmb2.12353 33488282 PMC7812476

[82] Aitken RJ , Clarkson JS . Cellular basis of defective sperm function and its association with the genesis of reactive oxygen species by human spermatozoa. Reproduction. 1987;81(2):459–469. 10.1530/jrf.0.0810459 2828610

[83] Aziz N , Saleh RA , Sharma RK , Lewis‐Jones I , Esfandiari N , Thomas AJ Jr , et al. Novel association between sperm reactive oxygen species production, sperm morphological defects, and the sperm deformity index. Fertil Steril. 2004;81(2):349–354. 10.1016/j.fertnstert.2003.06.026 14967372

[84] Yumura Y , Iwasaki A , Saito K , Ogawa T , Hirokawa M . Effect of reactive oxygen species in semen on the pregnancy of infertile couples. Int J Urol. 2009;16(2):202–207. 10.1111/j.1442-2042.2008.02213.x 19183232

[85] Yumura Y , Takeshima T , Kawahara T , Sanjo H , Sns K , Asai T , et al. Reactive oxygen species measured in the unprocessed semen samples of 715 infertile patients. Reprod Med Biol. 2017;16(4):354–363. 10.1002/rmb2.12052 29259489 PMC5715895

[86] Agarwal A , Parekh N , Selvam MKP , Henkel R , Shah R , Homa ST , et al. Male oxidative stress infertility (MOSI): proposed terminology and clinical practice guidelines for management of idiopathic male infertility. World J Men's Health. 2019;37(3):296–312. 10.5534/wjmh.190055 31081299 PMC6704307

[87] Takeshima T , Yumura Y , Yasuda K , Sanjo H , Kuroda S , Yamanaka H , et al. Inverse correlation between reactive oxygen species in unwashed semen and sperm motion parameters as measured by a computer‐assisted semen analyzer. Asian J Androl. 2017;19(3):350–354. 10.4103/1008-682x.173933 26975485 PMC5427793

[88] Ahelik A , Mändar R , Korrovits P , Karits P , Talving E , Rosenstein K , et al. Systemic oxidative stress could predict assisted reproductive technique outcome. J Assist Reprod Genet. 2015;32:699–704. 10.1007/s10815-015-0466-6 25813658 PMC4429443

[89] Simon L , Zini A , Dyachenko A , Ciampi A , Carrell DT . A systematic review and meta‐analysis to determine the effect of sperm DNA damage on in vitro fertilization and intracytoplasmic sperm injection outcome. Asian J Androl. 2017;19(1):80–90. 10.4103/1008-682x.182822 27345006 PMC5227680

[90] Tan J , Taskin O , Albert A , Bedaiwy MA . Association between sperm DNA fragmentation and idiopathic recurrent pregnancy loss: a systematic review and meta‐analysis. Reprod Biomed Online. 2019;38(6):951–960. 10.1016/j.rbmo.2018.12.029 30979611

[91] The ESHRE Guideline Group on RPL , Bender Atik R , Christiansen OB , Elson J , Kolte AM , Lewis S , et al. ESHRE guideline: recurrent pregnancy loss: an update in 2022. Hum Reprod Open. 2023;2023(1):hoad002. 10.1093/hropen/hoy004 36873081 PMC9982362

[92] Das S , Chattopadhyay R , Jana SK , Babu KN , Chakraborty C , Chakravarty B , et al. Cut‐off value of reactive oxygen species for predicting semen quality and fertilization outcome. Syst Biol Reprod Med. 2008;54(1):47–54. 10.1080/19396360701883274 18543865

[93] Aydemir B , Onaran I , Kiziler AR , Alici B , Akyolcu MC . The influence of oxidative damage on viscosity of seminal fluid in infertile men. J Androl. 2008;29(1):41–46. 10.2164/jandrol.107.003046 17673435

[94] Agarwal A , Majzoub A . Laboratory tests for oxidative stress. Indian J Urol. 2017;33(3):199–206. 10.4103/iju.iju_9_17 28717269 PMC5508430

[95] Aitken RJ . Free radicals, lipid peroxidation and sperm function. Reprod Fertil Dev. 1995;7(4):659–668. 10.1071/RD9950659 8711202

[96] McCord JM . The evolution of free radicals and oxidative stress. Am J Med. 2000;108(8):652–659. 10.1016/S0002-9343(00)00412-5 10856414

[97] Rael LT , Bar‐Or R , Mains CW , Slone DS , Levy AS , Bar‐Or D . Plasma oxidation‐reduction potential and protein oxidation in traumatic brain injury. J Neurotrauma. 2009;26(8):1203–1211. 10.1089/neu.2008.0816 19317602

[98] Agarwal A , Sharma R , Roychoudhury S , Du Plessis S , Sabanegh E . MiOXSYS: a novel method of measuring oxidation reduction potential in semen and seminal plasma. Fertil Steril. 2016;106(3):566–573. 10.1016/j.fertnstert.2016.05.013 27260688

[99] World Health Organization . WHO laboratory manual for the examination and processing of human semen. Geneva: World Health Organization; 2021.

[100] Walsh TJ , Schembri M , Turek PJ , Chan JM , Carroll PR , Smith JF , et al. Increased risk of high‐grade prostate cancer among infertile men. Cancer. 2010;116(9):2140–2147. 10.1002/cncr.25075 20309846 PMC2893877

[101] Agarwal A , Roychoudhury S , Bjugstad KB , Cho C‐L . Oxidation‐reduction potential of semen: what is its role in the treatment of male infertility? Ther Adv Urol. 2016;8(5):302–318. 10.1177/1756287216652779 27695529 PMC5004233

[102] Roychoudhury S , Sharma R , Sikka S , Agarwal A . Diagnostic application of total antioxidant capacity in seminal plasma to assess oxidative stress in male factor infertility. J Assist Reprod Genet. 2016;33:627–635. 10.1007/s10815-016-0677-5 26941096 PMC4870440

[103] Homa ST , Vassiliou AM , Stone J , Killeen AP , Dawkins A , Xie J , et al. A comparison between two assays for measuring seminal oxidative stress and their relationship with sperm DNA fragmentation and semen parameters. Genes. 2019;10(3):236. 10.3390/genes10030236 30893955 PMC6471935

[104] Agarwal A , Arafa M , Elbardisi H , Majzoub A , Alsaid S . Relationship between seminal oxidation reduction potential and sperm DNA fragmentation in infertile men. Fertil Steril. 2017;108(3):e316. 10.1016/j.fertnstert.2017.07.936

[105] Agarwal A , Roychoudhury S , Sharma R , Gupta S , Majzoub A , Sabanegh E . Diagnostic application of oxidation‐reduction potential assay for measurement of oxidative stress: clinical utility in male factor infertility. Reprod Biomed Online. 2017;34(1):48–57. 10.1016/j.rbmo.2016.10.008 27839743

[106] Schlegel PN , Sigman M , Collura B , De Jonge CJ , Eisenberg ML , Lamb DJ , et al. Diagnosis and treatment of infertility in men: AUA/ASRM guideline part I. J Urol. 2021;205(1):36–43. 10.1097/JU.0000000000001521 33295257

[107] Ferlin A , Calogero A , Krausz C , Lombardo F , Paoli D , Rago R , et al. Management of male factor infertility: position statement from the Italian Society of Andrology and Sexual Medicine (SIAMS) Endorsing Organization: Italian Society of Embryology, Reproduction, and Research (SIERR). J Endocrinol Invest. 2022;45(5):1085–1113. 10.1007/s40618-022-01741-6 35075609

[108] Acharya UR , Mishra M , Patro J , Panda MK . Effect of vitamins C and E on spermatogenesis in mice exposed to cadmium. Reprod Toxicol. 2008;25(1):84–88. 10.1016/j.reprotox.2007.10.004 18065194

[109] Angulo C , Maldonado R , Pulgar E , Mancilla H , Córdova A , Villarroel F , et al. Vitamin C and oxidative stress in the seminiferous epithelium. Biol Res. 2011;44(2):169–180. 10.4067/S0716-97602011000200009 22513420

[110] Cilio S , Rienzo M , Villano G , Mirto BF , Giampaglia G , Capone F , et al. Beneficial effects of antioxidants in male infertility management: a narrative review. Oxygen. 2022;2(1):1–11. 10.3390/oxygen2010001

[111] Alahmar AT , Calogero AE , Sengupta P , Dutta S . Coenzyme Q10 improves sperm parameters, oxidative stress markers and sperm DNA fragmentation in infertile patients with idiopathic oligoasthenozoospermia. World J Men's Health. 2021;39(2):346–351. 10.5534/wjmh.190145 32009311 PMC7994657

[112] Alahmar AT , Calogero AE , Singh R , Cannarella R , Sengupta P , Dutta S . Coenzyme Q10, oxidative stress, and male infertility: a review. Clin Exp Reprod Med. 2021;48(2):97–104. 10.5653/cerm.2020.04175 34078005 PMC8176150

[113] Kerns K , Zigo M , Sutovsky P . Zinc: a necessary ion for mammalian sperm fertilization competency. Int J Mol Sci. 2018;19(12):4097. 10.3390/ijms19124097 30567310 PMC6321397

[114] Martin‐Hidalgo D , Bragado MJ , Batista AR , Oliveira PF , Alves MG . Antioxidants and male fertility: from molecular studies to clinical evidence. Antioxidants. 2019;8(4):89. 10.3390/antiox8040089 30959797 PMC6523199

[115] Dutta S , Sengupta P , Roychoudhury S , Chakravarthi S , Wang CW , Slama P . Antioxidant paradox in male infertility:‘A blind eye’ on inflammation. Antioxidants. 2022;11(1):167. 10.3390/antiox11010167 35052671 PMC8772926

[116] Ali M , Martinez M , Parekh N . Are antioxidants a viable treatment option for male infertility? Andrologia. 2021;53(1):e13644. 10.1111/and.13644 32427374

[117] Sengupta P , Roychoudhury S , Nath M , Dutta S . Oxidative stress and idiopathic male infertility. In: Kesari KK , Roychoudhury S , editors. Oxidative stress and toxicity in reproductive biology and medicine: a comprehensive update on male infertility. Volume 1. Cham: Springer; 2022. p. 181–204. 10.1007/978-3-030-89340-8_9 35641871

[118] Arafa M , Agarwal A , Majzoub A , Panner Selvam MK , Baskaran S , Henkel R , et al. Efficacy of antioxidant supplementation on conventional and advanced sperm function tests in patients with idiopathic male infertility. Antioxidants. 2020;9(3):219. 10.3390/antiox9030219 32155908 PMC7139646

[119] Mongioi L , Calogero A , Vicari E , Condorelli R , Russo G , Privitera S , et al. The role of carnitine in male infertility. Andrology. 2016;4(5):800–807. 10.1111/andr.12191 27152678

[120] Zhou X , Liu F , Zhai S . Effect of L‐carnitine and/or L‐acetyl‐carnitine in nutrition treatment for male infertility: a systematic review. Asia Pac J Clin Nutr. 2007;16(1):383–390. 17392136

[121] Costa M , Canale D , Filicori M , D'lddio S , Lenzi A . L‐carnitine in idiopathic asthenozoospermia: a multicenter study. Italian Study Group on Carnitine and Male Infertility. Andrologia. 1994;26(3):155–159. 10.1111/j.1439-0272.1994.tb00780.x 8085668

[122] Micic S , Lalic N , Djordjevic D , Bojanic N , Bogavac‐Stanojevic N , Busetto GM , et al. Double‐blind, randomised, placebo‐controlled trial on the effect of L‐carnitine and L‐acetylcarnitine on sperm parameters in men with idiopathic oligoasthenozoospermia. Andrologia. 2019;51(6):e13267. 10.1111/and.13267 30873633 PMC6850469

[123] Schisterman EF , Sjaarda LA , Clemons T , Carrell DT , Perkins NJ , Johnstone E , et al. Effect of folic acid and zinc supplementation in men on semen quality and live birth among couples undergoing infertility treatment: a randomized clinical trial. JAMA. 2020;323(1):35–48. 10.1001/jama.2019.18714 31910279 PMC6990807

[124] Li X , Zeng Y‐m , He J , Luo B‐w , Lu X‐c , Zhu L‐l . Effects of folic acid and folic acid plus zinc supplements on the sperm characteristics and pregnancy outcomes of infertile men: a systematic review and meta‐analysis. Heliyon. 2023;9(7):e18224. 10.1016/j.heliyon.2023.e18224 37539255 PMC10395467

[125] Abd El‐Fadil Ibrahim H , Shalaby SI , Hebishy RMMA , Abdelfattah‐Hassan A , Abdel Ghani EMAM . Ameliorative effects of vitamin E and selenium on bleomycin‐induced male infertility. Slov Vet Res. 2023;60:433–438. 10.26873/SVR-1673-2023

[126] Bahmyari R , Ariafar A , Sayadi M , Hossieni S , Azima S . The effect of daily intake of selenium, vitamin E and folic acid on sperm parameters in males with idiopathic infertility: a single‐blind randomized controlled clinical trial. Int J Fertil Steril. 2021;15(1):8–14. 10.22074/ijfs.2021.6236 33497041 PMC7838762

[127] Alahmar AT , Sengupta P , Dutta S , Calogero AE . Coenzyme Q10, oxidative stress markers, and sperm DNA damage in men with idiopathic oligoasthenoteratospermia. Clin Exp Reprod Med. 2021;48(2):150–155. 10.5653/cerm.2020.04084 34078008 PMC8176152

[128] Agarwal A , Leisegang K , Majzoub A , Henkel R , Finelli R , Selvam MKP , et al. Utility of antioxidants in the treatment of male infertility: clinical guidelines based on a systematic review and analysis of evidence. World J Men's Health. 2021;39(2):233–290. 10.5534/wjmh.200196 33474843 PMC7994666

[129] Håkonsen LB , Thulstrup AM , Aggerholm AS , Olsen J , Bonde JP , Andersen CY , et al. Does weight loss improve semen quality and reproductive hormones? Results from a cohort of severely obese men. Reprod Health. 2011;8(1):24. 10.1186/1742-4755-8-24 21849026 PMC3177768

[130] Mir J , Franken D , Andrabi S , Ashraf M , Rao K . Impact of weight loss on sperm DNA integrity in obese men. Andrologia. 2018;50(4):e12957. 10.1111/and.12957 29388233

[131] Faure C , Dupont C , Baraibar MA , Ladouce R , Cedrin‐Durnerin I , Wolf JP , et al. In subfertile couple, abdominal fat loss in men is associated with improvement of sperm quality and pregnancy: a case‐series. PLoS One. 2014;9(2):e88956. 10.1371/journal.pone.0088956 24520319 PMC3919721

[132] McPherson NO , Lane M . Male obesity and subfertility, is it really about increased adiposity? Asian J Androl. 2015;17(3):450–458. 10.4103/1008-682x.148076 25652636 PMC4430951

[133] Karayiannis D , Kontogianni MD , Mendorou C , Douka L , Mastrominas M , Yiannakouris N . Association between adherence to the Mediterranean diet and semen quality parameters in male partners of couples attempting fertility. Hum Reprod. 2017;32(1):215–222. 10.1093/humrep/dew288 27994040

[134] Gaskins AJ , Colaci DS , Mendiola J , Swan SH , Chavarro JE . Dietary patterns and semen quality in young men. Hum Reprod. 2012;27(10):2899–2907. 10.1093/humrep/des298 22888168 PMC3442634

[135] Giahi L , Mohammadmoradi S , Javidan A , Sadeghi MR . Nutritional modifications in male infertility: a systematic review covering 2 decades. Nutr Rev. 2015;74(2):118–130. 10.1093/nutrit/nuv059 26705308 PMC4892303

[136] Du Plessis SS , Kashou A , Vaamonde D , Agarwal A . Is there a link between exercise and male factor infertility. Open Reprod Sci J. 2011;3:105–113. 10.2174/1874255601103010105

[137] Dai JB , Wang ZX , Qiao ZD . The hazardous effects of tobacco smoking on male fertility. Asian J Androl. 2015;17(6):954–960. 10.4103/1008-682x.150847 25851659 PMC4814952

[138] Harte CB , Meston CM . Association between smoking cessation and sexual health in men. BJU Int. 2012;109(6):888–896. 10.1111/j.1464-410X.2011.10503.x 21883852 PMC3235242

[139] Oyeyipo IP , Raji Y , Emikpe BO , Bolarinwa AF . Effects of nicotine on sperm characteristics and fertility profile in adult male rats: a possible role of cessation. J Reprod Infertil. 2011;12(3):201–207. 23926503 PMC3719292

[140] Sermondade N , Elloumi H , Berthaut I , Mathieu E , Delarouzière V , Ravel C , et al. Progressive alcohol‐induced sperm alterations leading to spermatogenic arrest, which was reversed after alcohol withdrawal. Reprod Biomed Online. 2010;20(3):324–327. 10.1016/j.rbmo.2009.12.003 20117050

[141] Gaur DS , Talekar MS , Pathak VP . Alcohol intake and cigarette smoking: impact of two major lifestyle factors on male fertility. Indian J Pathol Microbiol. 2010;53(1):35–40. 10.4103/0377-4929.59180 20090219

[142] Sansone A , Di Dato C , de Angelis C , Menafra D , Pozza C , Pivonello R , et al. Smoke, alcohol and drug addiction and male fertility. Reprod Biol Endocrinol. 2018;16(1):3. 10.1186/s12958-018-0320-7 29334961 PMC5769315

[143] Jensen TK , Swan SH , Skakkebæk NE , Rasmussen S , Jørgensen N . Caffeine intake and semen quality in a population of 2,554 young Danish men. Am J Epidemiol. 2010;171(8):883–891. 10.1093/aje/kwq007 20338976

[144] Du Plessis SS , Agarwal A , Syriac A . Marijuana, phytocannabinoids, the endocannabinoid system, and male fertility. J Assist Reprod Genet. 2015;32(11):1575–1588. 10.1007/s10815-015-0553-8 26277482 PMC4651943

[145] McBride JA , Coward RM . Recovery of spermatogenesis following testosterone replacement therapy or anabolic‐androgenic steroid use. Asian J Androl. 2016;18(3):373–380. 10.4103/1008-682x.173938 26908067 PMC4854084

[146] Bhongade M , Prasad S , Jiloha R , Ray P , Mohapatra S , Koner B . Effect of psychological stress on fertility hormones and seminal quality in male partners of infertile couples. Andrologia. 2015;47(3):336–342. 10.1111/and.12268 24673246

[147] Yao DF , Mills JN . Male infertility: lifestyle factors and holistic, complementary, and alternative therapies. Asian J Androl. 2016;18(3):410. 26952957 10.4103/1008-682X.175779 PMC4854092

[148] Viganò P , Chiaffarino F , Bonzi V , Salonia A , Ricci E , Papaleo E , et al. Sleep disturbances and semen quality in an Italian cross sectional study. Basic Clin Androl. 2017;27(1):16. 10.1186/s12610-017-0060-0 28835845 PMC5563907

[149] Alvarenga TA , Hirotsu C , Mazaro‐Costa R , Tufik S , Andersen ML . Impairment of male reproductive function after sleep deprivation. Fertil Steril. 2015;103(5):1355–1362.e1. 10.1016/j.fertnstert.2015.02.002 25747127

[150] Calogero AE , Cannarella R , Agarwal A , Hamoda TAA , Rambhatla A , Saleh R , et al. The renaissance of male infertility management in the golden age of andrology. World J Men's Health. 2023;41(2):237–254. 10.5534/wjmh.220213 36649928 PMC10042649

[151] Barati E , Nikzad H , Karimian M . Oxidative stress and male infertility: current knowledge of pathophysiology and role of antioxidant therapy in disease management. Cell Mol Life Sci. 2020;77:93–113. 10.1007/s00018-019-03253-8 31377843 PMC11105059

[152] Agarwal A , Finelli R , Selvam MKP , Leisegang K , Majzoub A , Tadros N , et al. A global survey of reproductive specialists to determine the clinical utility of oxidative stress testing and antioxidant use in male infertility. World J Men's Health. 2021;39(3):470–488. 10.5534/wjmh.210025 33831977 PMC8255391

[153] Lu Y , Oura S , Matsumura T , Oji A , Sakurai N , Fujihara Y , et al. CRISPR/Cas9‐mediated genome editing reveals 30 testis‐enriched genes dispensable for male fertility in mice. Biol Reprod. 2019;101(2):501–511. 10.1093/biolre/ioz103 31201419 PMC6735960

[154] Sánchez‐Rubio F , Soria‐Meneses PJ , Jurado‐Campos A , Bartolomé‐García J , Gómez‐Rubio V , Soler AJ , et al. Nanotechnology in reproduction: vitamin E nanoemulsions for reducing oxidative stress in sperm cells. Free Radic Biol Med. 2020;160:47–56. 10.1016/j.freeradbiomed.2020.07.024 32768571

[155] Raeeszadeh M , Karimfar B , Amiri AA , Akbari A . Protective effect of nano‐vitamin C on infertility due to oxidative stress induced by lead and arsenic in male rats. J Chem. 2021;2021:1–12. 10.1155/2021/9589345

[156] Helli B , Kavianpour M , Ghaedi E , Dadfar M , Haghighian HK . Probiotic effects on sperm parameters, oxidative stress index, inflammatory factors and sex hormones in infertile men. Hum Fertil. 2022;25(3):499–507. 10.1080/14647273.2020.1824080 32985280

[157] Panner Selvam MK , Finelli R , Agarwal A , Henkel R . Proteomics and metabolomics—current and future perspectives in clinical andrology. Andrologia. 2021;53(2):e13711. 10.1111/and.13711 32598566

[158] Gosalvez J , Tvrda E , Agarwal A . Free radical and superoxide reactivity detection in semen quality assessment: past, present, and future. J Assist Reprod Genet. 2017;34(6):697–707. 10.1007/s10815-017-0912-8 28341974 PMC5445049

[159] Aitken RJ . Impact of oxidative stress on male and female germ cells: implications for fertility. Reproduction. 2020;159(4):R189–R201. 10.1530/rep-19-0452 31846434

[160] Sengupta P , Dutta S , Irez T . Oxidants and antioxidants in male reproduction: roles of oxidative and reductive stress. J Integr Sci Technol. 2024;12(3):753–762. 10.62110/sciencein.jist.2024.v12.753

[161] Ayad B , Omolaoye TS , Louw N , Ramsunder Y , Skosana BT , Oyeipo PI , et al. Oxidative stress and male infertility: evidence from a research perspective. Front Reprod Health. 2022;4:822257. 10.3389/frph.2022.822257 36303652 PMC9580735

[162] Henkel RR . Leukocytes and oxidative stress: dilemma for sperm function and male fertility. Asian J Androl. 2011;13(1):43–52. 10.1038/aja.2010.76 21076433 PMC3739401

[163] Aitken RJ , Smith TB , Jobling MS , Baker MA , De Iuliis GN . Oxidative stress and male reproductive health. Asian J Androl. 2014;16(1):31–38. 10.4103/1008-682x.122203 24369131 PMC3901879

[164] Castleton P , Gyawali P , Mathews N , Mutuku SM , Sharkey DJ , McPherson NO . MiOXSYS® and OxiSperm® II assays appear to provide no clinical utility for determining oxidative stress in human sperm—results from repeated semen collections. Andrology. 2023;11(8):1566–1578. 10.1111/andr.13356 36455546

[165] Agarwal A , Henkel R , Sharma R , Tadros N , Sabanegh E . Determination of seminal oxidation–reduction potential (ORP) as an easy and cost‐effective clinical marker of male infertility. Andrologia. 2018;50(3):e12914. 10.1111/and.12914 29057493

[166] Tunc O , Thompson J , Tremellen K . Development of the NBT assay as a marker of sperm oxidative stress. Int J Androl. 2010;33(1):13–21. 10.1111/j.1365-2605.2008.00941.x 19076253

[167] Hussain T , Kandeel M , Metwally E , Murtaza G , Kalhoro DH , Yin Y , et al. Unraveling the harmful effect of oxidative stress on male fertility: a mechanistic insight. Front Endocrinol. 2023;14:1070692. 10.3389/fendo.2023.1070692 PMC9968806 36860366

